# Clinical applicability and cost of a 46-gene panel for genomic analysis of solid tumours: Retrospective validation and prospective audit in the UK National Health Service

**DOI:** 10.1371/journal.pmed.1002230

**Published:** 2017-02-14

**Authors:** Angela Hamblin, Sarah Wordsworth, Jilles M. Fermont, Suzanne Page, Kulvinder Kaur, Carme Camps, Pamela Kaisaki, Avinash Gupta, Denis Talbot, Mark Middleton, Shirley Henderson, Anthony Cutts, Dimitrios V. Vavoulis, Nick Housby, Ian Tomlinson, Jenny C. Taylor, Anna Schuh

**Affiliations:** 1 Molecular Diagnostics Centre, Oxford University Hospitals NHS Foundation Trust, Oxford, United Kingdom; 2 National Institute for Health Research Biomedical Research Centre, Oxford, United Kingdom; 3 Health Economics Research Centre, Nuffield Department of Population Health, University of Oxford, Oxford, United Kingdom; 4 Experimental Medicine and Immunotherapeutics, Department of Medicine, University of Cambridge, Cambridge, United Kingdom; 5 Wellcome Trust Centre for Human Genetics, University of Oxford, Oxford, United Kingdom; 6 Department of Oncology, University of Oxford, Oxford, United Kingdom; MSKCC, UNITED STATES

## Abstract

**Background:**

Single gene tests to predict whether cancers respond to specific targeted therapies are performed increasingly often. Advances in sequencing technology, collectively referred to as next generation sequencing (NGS), mean the entire cancer genome or parts of it can now be sequenced at speed with increased depth and sensitivity. However, translation of NGS into routine cancer care has been slow. Healthcare stakeholders are unclear about the clinical utility of NGS and are concerned it could be an expensive addition to cancer diagnostics, rather than an affordable alternative to single gene testing.

**Methods and findings:**

We validated a 46-gene hotspot cancer panel assay allowing multiple gene testing from small diagnostic biopsies. From 1 January 2013 to 31 December 2013, solid tumour samples (including non-small-cell lung carcinoma [NSCLC], colorectal carcinoma, and melanoma) were sequenced in the context of the UK National Health Service from 351 consecutively submitted prospective cases for which treating clinicians thought the patient had potential to benefit from more extensive genetic analysis. Following histological assessment, tumour-rich regions of formalin-fixed paraffin-embedded (FFPE) sections underwent macrodissection, DNA extraction, NGS, and analysis using a pipeline centred on Torrent Suite software. With a median turnaround time of seven working days, an integrated clinical report was produced indicating the variants detected, including those with potential diagnostic, prognostic, therapeutic, or clinical trial entry implications. Accompanying phenotypic data were collected, and a detailed cost analysis of the panel compared with single gene testing was undertaken to assess affordability for routine patient care.

Panel sequencing was successful for 97% (342/351) of tumour samples in the prospective cohort and showed 100% concordance with known mutations (detected using cobas assays). At least one mutation was identified in 87% (296/342) of tumours. A locally actionable mutation (i.e., available targeted treatment or clinical trial) was identified in 122/351 patients (35%). Forty patients received targeted treatment, in 22/40 (55%) cases solely due to use of the panel. Examination of published data on the potential efficacy of targeted therapies showed theoretically actionable mutations (i.e., mutations for which targeted treatment was potentially appropriate) in 66% (71/107) and 39% (41/105) of melanoma and NSCLC patients, respectively. At a cost of £339 (US$449) per patient, the panel was less expensive locally than performing more than two or three single gene tests.

Study limitations include the use of FFPE samples, which do not always provide high-quality DNA, and the use of “real world” data: submission of cases for sequencing did not always follow clinical guidelines, meaning that when mutations were detected, patients were not always eligible for targeted treatments on clinical grounds.

**Conclusions:**

This study demonstrates that more extensive tumour sequencing can identify mutations that could improve clinical decision-making in routine cancer care, potentially improving patient outcomes, at an affordable level for healthcare providers.

## Abstract 2

Sarah Wordsworth and colleagues study the feasibility of employing next-generation sequencing of tumor DNA in clinical practice.

## Author summary

**Why was this study done?:**

- Healthcare planners and oncologists require real world evidence that next generation sequencing (NGS) technologies improve gene mutation detection and enable more appropriate use of targeted drug therapies.
- With a range of genomic testing options available for cancer patients, we need to know whether healthcare systems can afford to implement cancer panels in routine clinical care, even if they are effective.

**What did the researchers do and find?:**

- This study assessed a 46-gene hotspot cancer panel assay allowing multiple gene testing of small diagnostic cancer biopsies in the context of the UK National Health Service. Tumour samples (including non-small-cell lung cancer, melanoma, and colorectal carcinoma) from 351 patients who treating clinicians thought might benefit from more extensive genetic analysis underwent NGS using the panel. A clinical report was produced with a median turnaround time of seven working days that indicated all mutations detected, including those with potential diagnostic, prognostic, therapeutic, or clinical trial entry implications.
- Clinical data were collected for patients whose tumour samples underwent sequencing in order to assess changes to clinical management resulting from this test.
- An accompanying detailed cost analysis was performed to determine the affordability of the panel compared to existing single gene testing options.
- The panel demonstrated at least one mutation in 87% (296/342) of successfully sequenced tumours.
- Forty patients in this cohort received targeted treatments on the basis of genetic data obtained using the panel. For 22 of these patients, there was no alternative genetic test available locally to produce this data.
- Mutation detection with the panel costs £339 (US$449) per patient, compared with single gene testing ranging from £71 to £141 (US$94–US$187) per test, depending on the mutation type. If more than two or three genes are examined (depending on the cancer type), using the panel is less expensive than single gene testing.

**What do these findings mean?:**

- The panel assay is a useful method to identify genetic mutations in tumours that can extend the range of therapeutic options available to patients.
- In terms of costs and affordability, the panel may be a justifiable option if 2–3 or more genes need to be examined.
- Further data need to be collected on the clinical outcomes of patients accessing drugs as a result of more extensive sequencing data outside the scope of single gene/mutation tests.
- In addition to supporting routine clinical care, the panel can be used to support research studies where treatment choices are genetically determined.

## Introduction

Historically, the standard approach to testing for somatic mutations in cancers has been single gene testing using methods such as Sanger sequencing. With such methods, candidate genes are examined for mutations, and, as a result, patients may become eligible to enter a clinical trial or receive targeted drug therapies [1–3]. Advances in sequencing technology, collectively referred to as next generation sequencing (NGS), mean that the entire cancer genome (whole genome sequencing [WGS]) or parts of it (via targeted panels or whole exome sequencing [WES]) can now be sequenced in hours and at great depth and increasing sensitivity. However, while NGS offers high-throughput, rapid, and accurate testing of multiple genes, it remains to be proven whether it also leads to more appropriate use of targeted drug therapies and an enhanced ability to identify patients who are more likely to benefit from treatment compared with single gene sequencing.

An increasing number of primarily privately funded laboratories are already using NGS to profile tumours for mutations in multiple cancer genes simultaneously, with designs ranging from hotspot panels to a 287-gene panel covering all exons of constituent genes and selected introns (those involved in translocations). DNA requirements vary from 10 to 750 ng, and sample types evaluated include fresh frozen tissue, formalin-fixed paraffin-embedded (FFPE) samples, and fine needle aspirate specimens [4–7]. Many NGS technologies have demonstrated good sensitivity and excellent correlation with standard genetic techniques, as well as providing potentially clinically actionable information. However, the widespread translation of NGS technologies into routine cancer diagnostics has been slow due to technical obstacles (e.g., problems with robust bioinformatics) compromising clinical-grade validation, challenges with clinical interpretation (e.g., paucity of functional data), the absence of genotype–phenotype databases for cancer [8], a lack of demonstrable clinical utility surpassing that of single gene testing, and concern over the costs of NGS to healthcare payers.

Healthcare systems are now recognising the need to understand how to efficiently use genomic technologies in the context of precision medicine and verify their safety and effectiveness with timely evidence [9]. However, there is limited empirical evidence on whether results obtained from NGS technology direct clinical management and/or improve patient outcomes and whether they represent an efficient use of healthcare resources or are just an expensive addition to cancer care.

The aim of our study was to investigate whether a targeted hotspot NGS cancer panel could be translated into routine patient care in the UK National Health Service (NHS). This study involved a clinical-grade optimisation and validation of the panel and bioinformatics pipeline for diagnostics, an assessment of the panel’s impact on clinical management, and a cost analysis of the panel compared with single gene testing.

## Methods

### Study ethics

Clinical consent was obtained for all samples prior to genetic panel testing. The validation cohort included samples from the VICTOR trial that were consented for genetic analysis (approval obtained from Oxford Research Ethics Committee B [approval number 05\Q1605\66]). For the prospective cohort analysis, we used anonymised diagnostic samples for which ethical approval for service development was not required.

### Study design

**Fig 1:**
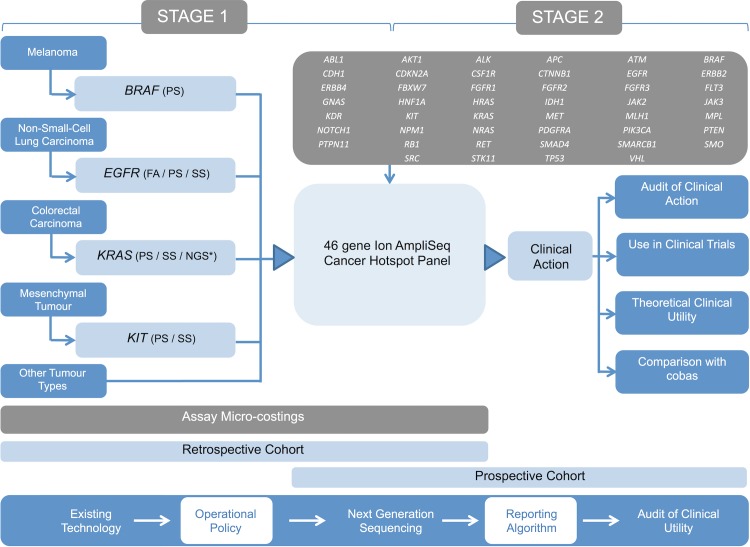
Study design. Outline of the study design demonstrating the existing genetic testing repertoire of the laboratory and the proposed NGS assay (cancer panel). Stage 1 involved the technical validation of the panel using a retrospective cohort of samples and performance of micro-costings. Stage 2 involved the panel’s introduction into diagnostic pathways using a prospective patient cohort. An operational policy was developed to select samples for routine analysis using the panel, and comprehensive phenotypic data were obtained in order to assess impact on clinical management. Tumour-appropriate tandem analysis of *BRAF*, *EGFR*, and *KRAS* was performed using cobas assays. FA, fragment analysis; NGS*, alternative next generation sequencing; PS, pyrosequencing; SS, Sanger sequencing.

The NGS technology we assessed was the Ion AmpliSeq Cancer Hotspot Panel (Thermo Fisher Scientific; 46 genes, 189 amplicons). This study was completed in two stages. Stage 1 involved technical validation of the panel using an anonymised retrospective cohort of previously genotyped tumour samples (undertaken as a service development) and comparative costings of the assay with existing technologies in use. Stage 2 was clinical implementation of the validated panel with an accompanying prospective audit of the clinical impact of this assay on treatment choice. Study design, including the genes partially covered by the panel, is presented in Fig 1.

### Patient cohorts and data collection

The retrospective cohort used for technical validation (*n =* 108) was composed of two sequentially tested groups; cohort 1 (*n =* 63) and cohort 2 (*n =* 45). Cohort 1 included samples from colorectal carcinoma (CRC), non-small-cell lung cancer (NSCLC), melanoma, and gastrointestinal stromal tumour (GIST) patients that were tested in tandem with standard diagnostic assays (S1 Text; S1 Table). Cohort 2 included previously sequenced CRC samples from the VICTOR (Vioxx In Colorectal cancer Therapy: definition of Optimal Regime) trial [10] as well as NSCLC and GIST specimens. Cohort 1 provided a more diverse range of tumour types, whilst cohort 2 provided mutational information on a wider range of genes than was available from standard diagnostic assays.

The prospective cohort (*n =* 351) consisted of malignant specimens (predominantly mesenchymal tumours, melanoma, NSCLC, and CRC) consecutively submitted to the Oxford Molecular Diagnostics Centre over a 12-mo period. The decision to submit a tumour sample for testing was made at the weekly multidisciplinary team meeting, with input from the treating oncologist and reporting histopathologist. An operational policy with sample testing algorithms, designed to ensure that testing was restricted to those patients with the potential to benefit from the acquisition of more comprehensive sequencing data, was available to provide guidance on appropriate samples for testing (S1 Fig). This cohort was also used to inform the translation of the panel into routine NHS care.

Phenotypic data were obtained for patients in the prospective cohort. In addition to tumour type, number of mutations, and drugs given, socio-demographic data were collected according to the best practice guidance for clinical audit [11]. In order to evaluate turnaround times, the date of assay request was also collected.

### Sample preparation and conventional analysis

**Fig 2:**
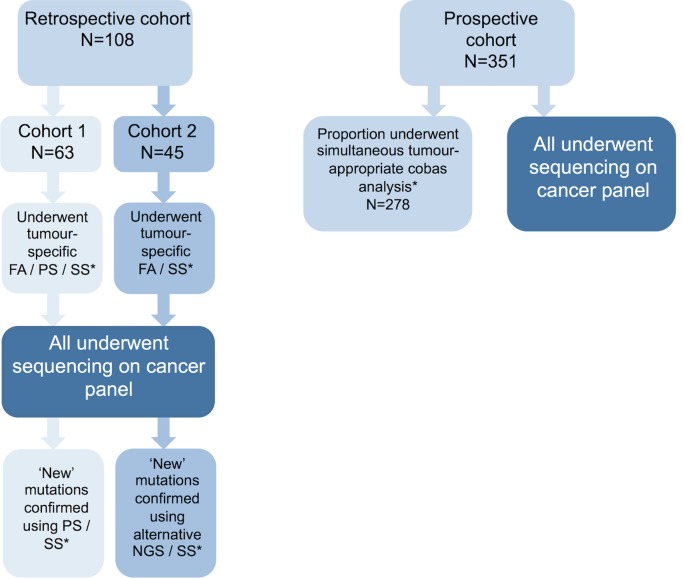
Patient cohort details. Description of the patient cohorts used in this study. Retrospective cohorts 1 and 2 were used in stage 1 of this study, while the prospective patient cohort was used for stage 2. Retrospective cohort samples had tumour-type-appropriate conventional diagnostic mutation screening prior to testing with the panel. Any novel variants detected by the panel were confirmed using an alternative assay. Prospective cohort samples had tandem analysis of tumour-appropriate genes using cobas assays. *See S1 Table for details of assay and genes tested in each cohort. FA, fragment analysis; NGS; next generation sequencing; PS, pyrosequencing; SS, Sanger sequencing.

FFPE samples from both patient cohorts were macrodissected and DNA extraction performed as described in S1 Text. All retrospective cohort samples had conventional diagnostic testing performed in tandem, e.g., Sanger sequencing, pyrosequencing, and fragment analysis depending on sample type and gene under investigation. Among the prospective cohort, 278/351 samples had tandem cobas (Roche Diagnostics) analysis performed as per DNA availability and referring clinician preference, allowing comparison of the two alternative technologies. Cohort design and testing strategy are outlined in Fig 2 and S1 Table.

### Next generation sequencing

The design of the panel (i.e., which genes, exons, and codons were included) was based on variants for which there were potential therapeutic, prognostic, or diagnostic implications (for full details see S2 Table). Variants with therapeutic options included both those with established treatments approved by the US Food and Drug Administration/European Medicines Agency (e.g., *BRAF*, *KRAS*, and *EGFR*) and those potentially targetable (e.g., *IDH1*). The breadth of variants covered by the assay in any gene was designed to extend that available via single gene tests (e.g., inclusion of *KRAS* exon 5 to cover codon [12]). The overall scope (i.e., number of targets covered by the panel) was a balance between providing maximal data of clinical utility (i.e., potentially actionable) and permitting sufficient multiplexing of samples on a single sequencing chip to render the assay affordable.

DNA from all samples in both cohorts underwent NGS using the Ion AmpliSeq Cancer Hotspot Panel (Thermo Fisher Scientific). Ten nanograms of tumour DNA was amplified using the Ion AmpliSeq Library Kit 2.0 and Ion AmpliSeq Cancer Primer Pool, designed to detect mutations in hotspots in 46 genes (Fig 1), and indexed using the Ion Xpress DNA Barcode Adaptor 1–96 Kit (all Thermo Fisher Scientific) according to the manufacturer’s instructions. Libraries were purified using the AxyPrep Mag PCR Clean-Up Kit (Axygen Biosciences) and quantified using either an Agilent 2100 Bioanalyzer with the DNA High Sensitivity Kit (both Agilent Technologies) or quantitative PCR with the Ion Library Quantitation Kit (Thermo Fisher Scientific). Individual amplified libraries were diluted to 20 pM, and four or eight libraries were multiplexed to give a final concentration of 20 pM. Template-positive Ion Sphere Particles containing clonally amplified DNA were prepared using the Ion OneTouch Template Kit v2 and enriched using the Ion OneTouch ES as per manufacturer’s instructions. Libraries were sequenced on the Ion Torrent Personal Genome Machine with four and eight barcoded samples multiplexed on 316 and 318 chips, respectively (all Thermo Fisher Scientific).

### Next generation sequencing data analysis and clinical reporting

**Fig 3:**
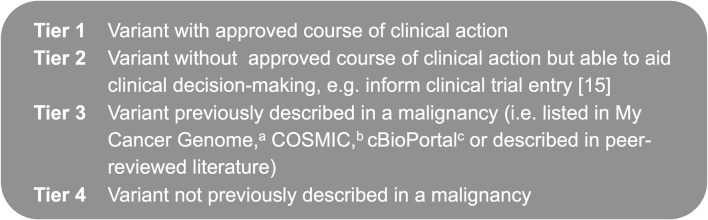
Variant classification tiers. Tier system used to classify variants detected using the cancer panel. ^a^My Cancer Genome: http://www.mycancergenome.org. ^b^COSMIC (Catalogue of Somatic Mutations in Cancer): http://cancer.sanger.ac.uk/cancergenome/projects/cosmic. ^c^cBioPortal for Cancer Genomics: http://www.cbioportal.org/public-portal/.

Sequencing data were analysed using Torrent Suite software, optimised in an iterative fashion (S1 Text). In a similar fashion to other investigators, we used a tier system to classify variants [13,14] (Fig 3), with all being reported to clinicians in an integrated molecular and histopathological report overseen by a senior clinical scientist and histopathologist.

**Fig 4:**
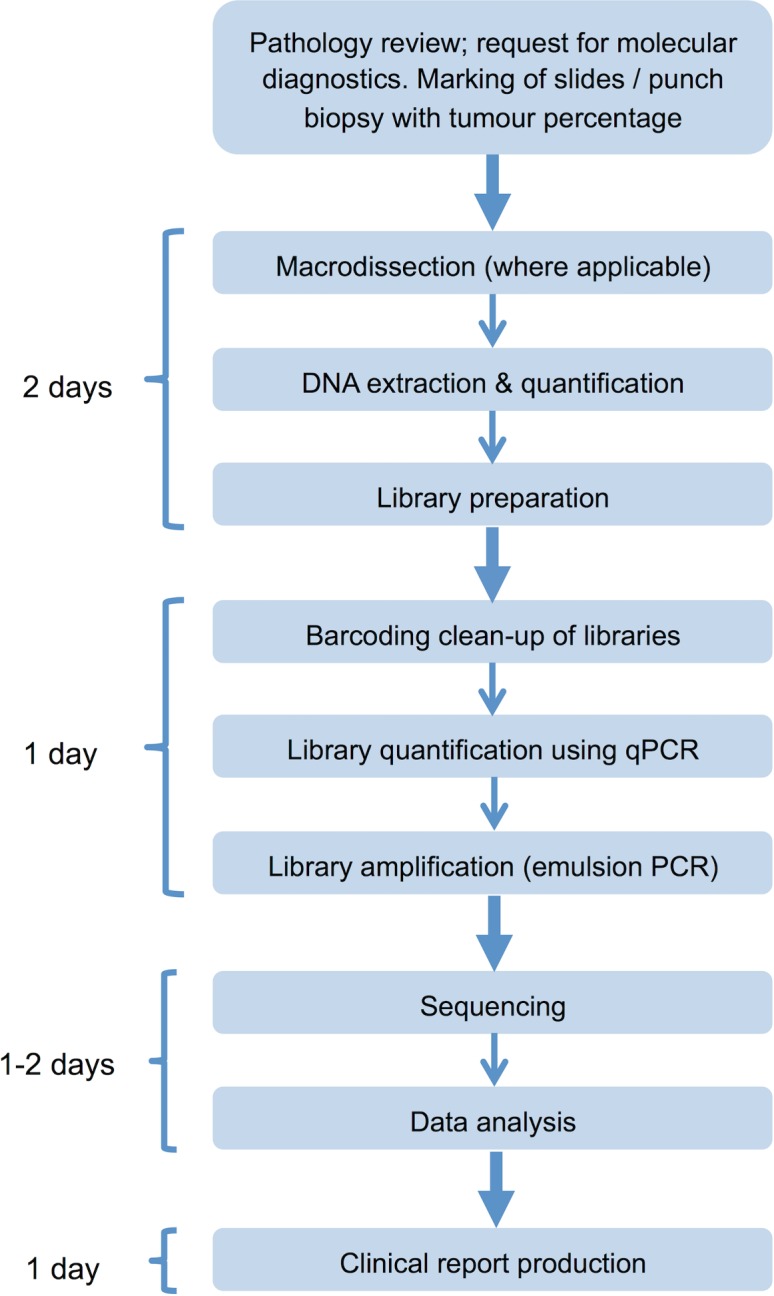
Next generation sequencing laboratory workflow. Assay workflow indicating time requirement for each stage of the process; clinical reports can be produced within 5–6 d of receipt of the tumour sample in the laboratory. qPCR, quantitative PCR.

The panel was integrated into diagnostic laboratory workflows to enable reporting within a clinically relevant timescale (Fig 4).

### Cost analysis

We undertook a detailed micro-costing of both the panel and the cobas system at the Oxford Molecular Diagnostics Centre to determine whether NGS was likely to be sufficiently affordable to translate into routine care. Micro-costing is a highly detailed costing approach that identifies all the underlying resources required for an intervention/activity, such as equipment, consumables, and staff time, and then calculates costs for these resources. The standard operating procedures for the alternative technologies were used to develop costing questionnaires to collect the resource use information (S2 Text). The questionnaires covered each stage in the experimental protocol from sample preparation to data interpretation and reporting. Resource information on staff time, consumables, and equipment was derived from the questionnaires. We accounted for the expected cost of errors during the testing processes. For most equipment items, the cost was spread over the item’s predicted lifetime and depreciated using equivalent annual costing with a discount rate of 3.5%. The cost of the cobas z 480 Analyzer is covered in a combined cost with the mutation kits by the machine manufacturer Roche Diagnostics. The costs of reagents were obtained from prices reported by the diagnostics laboratory and also by contacting reagent manufacturers. Commercially available, rather than any discounted prices, were used where possible. Price per sample was based on the measured yearly throughput of the sequencing platforms, which was 832 (clinical and research samples, based on sequencing 16 samples per week for 52 wk) for the Ion AmpliSeq panel and 2,340 (45 samples per week) for the cobas.

To compare the panel costs with single gene test costs, we used NSCLC, melanoma, and CRC as examples, because these cancers made up the majority of cases in our clinical study. Several alternative costing scenarios were costed, based on clinical practice and the UK National External Quality Assessment Service (UK NEQAS) 2013 guidelines for molecular pathology [15]. For example, for NSCLC we compared the following testing scenarios: (a) the Ion AmpliSeq panel, (b) cobas with single and multiple mutation kits (*EGFR*, *BRAF*, and *KRAS*), and (c) cobas with an *EGFR* mutation kit, followed by the Ion AmpliSeq panel. Scenario (c) was included to confirm with the Ion AmpliSeq panel whether a lung cancer that was *EGFR* negative was also *KRAS* and *BRAF* negative: if all three genes tested negative, patients went on to have *ALK* testing, as these mutations have been found to be mutually exclusive [16].

## Results

### Validation of the cancer panel

**Fig 5:**
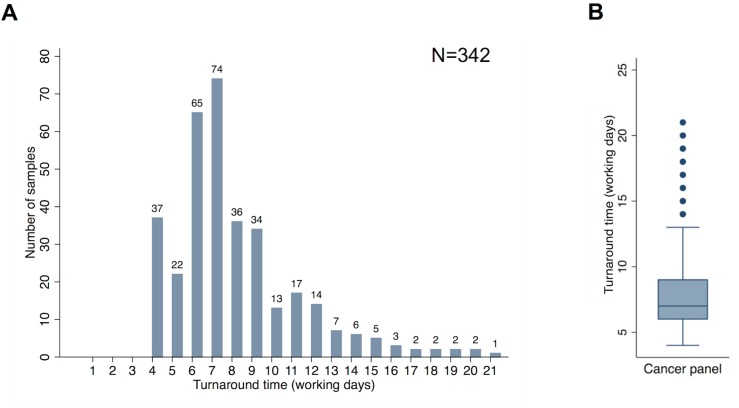
Turnaround times. (A) Distribution of turnaround time from request of the panel assay to production of a clinical report for samples in the prospective cohort. (B) Median, interquartile range, and outlying turnaround times from request of the panel assay to production of a clinical report for samples in the prospective cohort. Analysis and figure generation performed using Stata version 13 (StataCorp).

Detailed information concerning the technical validation of the panel is provided in S1 Text. Among the prospective cohort, comparative failure rates of the panel and cobas were examined, as were real world turnaround times. The overall panel failure rate in this cohort was 2.6% (9/351). Among those samples analysed using both the panel and cobas, the failure rates were comparable: 0.7% (2/278) and 1.1% (3/278). Fig 5 demonstrates the range of turnaround times (in working days) observed for the panel (*n =* 342). The median turnaround time was seven working days, with an interquartile range of 6–9 d.

Turnaround time represents the timespan from assay request in the laboratory to report generation. It should be noted that this does not include the time taken to make the original histopathological diagnosis or produce sections or punches of the tumour suitable for DNA extraction. Turnaround times of 4 d were observed when extracted DNA was already available within the laboratory (due to prior single gene tests having been previously requested). Longer turnaround times could be accounted for by occasional failed assays being repeated, the need to multiplex samples on the sequencing chip, and the fact that the assay was performed only once a week, meaning that if a sample arrived immediately after the assay was initiated, the sample had a wait of five working days before library preparation was commenced.

Although turnaround times of 2–3 working days from sample receipt in the laboratory to report generation were possible for single gene cobas tests, the laboratory practice of batching samples for this analysis and performing assays on a weekly basis meant three sequential single gene tests had a turnaround time in excess of ten working days.

### Patient demographics and mutations detected

The 351 prospective cohort samples sequenced had comprehensive phenotype data, a summary of which is given in Table 1; 52% were female, and the median age was 68 y (range 9–95) at the time of tumour sampling. The predominant tumour types tested were melanoma (31.1%), NSCLC (30.8%), and CRC (25.1%), all of which should have been metastatic or unresectable, correlating with the malignancies for which there are targeted therapies approved by the National Institute for Health and Care Excellence (NICE) (S7 Table) [1–3,17]. The remaining samples were mostly mesenchymal tumours, e.g., GISTs, (for which tyrosine kinase inhibitors [TKIs] may be appropriate), or were submitted to allow assessment for clinical trial entry [18].

**Table 1:** Prospective cohort patient characteristics (*n* = 351 patients).

Patient Characteristic	Subcategory	Value
**Testing method**	Ion AmpliSeq panel (Thermo Fisher Scientific)	351 (100%)
	cobas (Roche Diagnostics)	278 (79%)
**Gender**	Male	168 (48%)
	Female	181 (52%)
	Missing	2
**Age when sample taken**	Median (range) (years)	68 (9–95)
	Missing	5
**Pathology**	**Melanoma**	109 (31%)
	*BRAF* mutation	41 (38%)
	*NRAS* mutation	38 (35%)
	*KIT* mutation	4 (4%)
	**Non-small-cell lung cancer**	108 (31%)
	*EGFR* mutation	21 (19%)
	*BRAF* mutation	11 (10%)
	*KRAS* mutation	26 (24%)
	*PIK3CA* mutation	4 (4%)
	**Colorectal carcinoma**	88 (25%)
	*KRAS* mutation	37 (42%)
	**Other (e.g., GIST and breast cancer)**	46 (13%)
**Number of mutations found**	0	46 (13%)
	1	131 (37%)
	2	98 (28%)
	3	44 (13%)
	4	19 (5%)
	5	3 (1%)
	6	1 (0%)
	Test failed	9 (3%)
**Type of treatment**	Chemotherapy	91 (26%)
	Targeted treatment	53 (15%)
	No drug treatment	207 (59%)

Values are number of patients (percent) unless otherwise indicated.

GIST, gastrointestinal stromal tumour.

**Fig 6:**
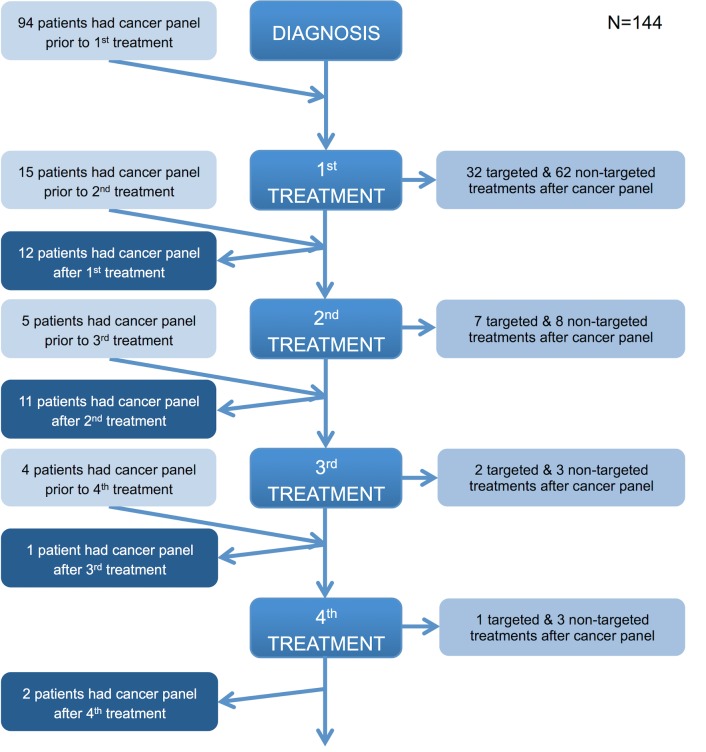
Timing of the cancer panel in the patient care pathway. The flowchart illustrates when the panel was performed in the patient journey relative to diagnosis and lines of treatment and the type of treatment (if any) subsequently given. If a patient received a line(s) of treatment after the panel was performed, they were considered to have had the panel prior to a treatment. If a patient did not receive any further lines of treatment after the panel was performed, they were considered to have had the panel after a treatment. Those patients who did not receive any treatment or for whom there are no details of any treatment received are not represented in the flowchart. All patients in the prospective cohort had their tumour analysed on a single occasion.

In all, 144/351 (41.0%) patients received pharmacological therapy, either chemotherapy or targeted molecular therapy. Treatment decisions were determined at the tumour-specific multidisciplinary team meeting and followed locally endorsed guidelines in line with national and international recommendations for each tumour type. Targeted therapies were administered in accordance with NICE guidelines [1–3,17] or in the context of a clinical trial. Fig 6 demonstrates the timing of the assay in the treatment pathway of these 144 patients. All patients presented in this study had a single sample analysed once using the panel; most often, the assay was performed prior to any treatment, with only a few patients having the test done after three or more lines of therapy, usually to facilitate clinical trial entry (e.g., a trial of a PI3K inhibitor in breast cancer [19]). The proportions of these 144 pharmacologically treated patients receiving targeted and non-targeted therapies were 36.8% (53/144) and 63.2% (91/144), respectively.

**Fig 7:**
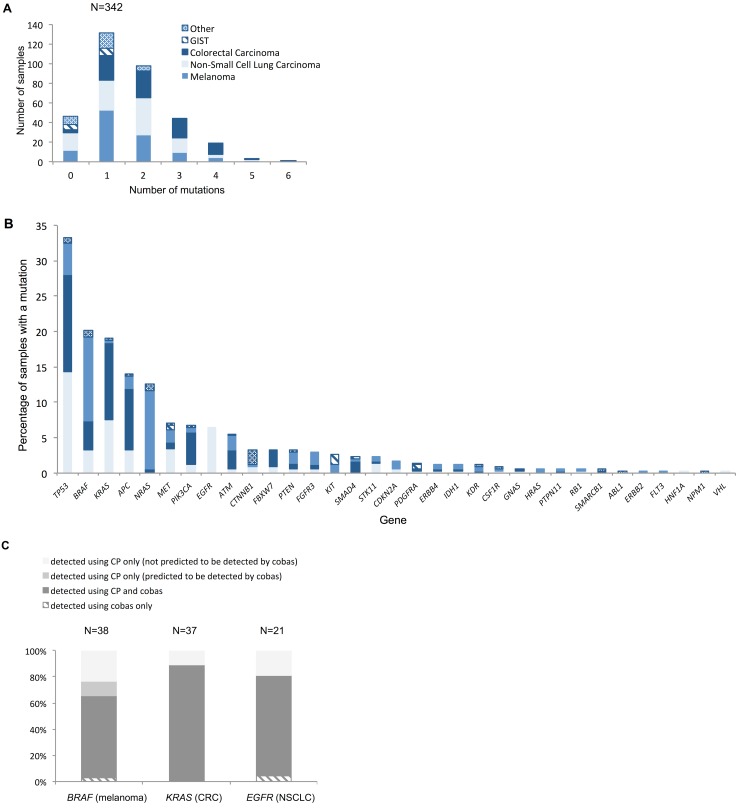
Distribution of mutations. (A) Number of mutations per histological sample by tumour type. Across all tumour types, mean and median numbers of mutations detected were 1.63 and 1, respectively. The hierarchy of least to most mutated tumour type was GIST, other, melanoma, NSCLC, and CRC, with mean/median numbers of mutations of 0.71/1, 0.97/1, 1.49/1, 1.61/2, and 2.17/2, respectively. (B) Distribution of mutations across the different genes represented on the panel for the different tumour types. The key in (A) applies to both charts. For genes that are not displayed in (B), no mutations were detected. (C) Percentage of mutations in key clinically actionable genes detected by standard diagnostic methods and the panel in the prospective cohort (*N* relates to number of mutations). Only samples that had tandem tumour-appropriate cobas analysis are included in this comparison. CP, cancer panel; CRC, colorectal carcinoma; GIST, gastrointestinal stromal tumour; NSCLC, non-small-cell lung carcinoma.

Fig 7 demonstrates the number of mutations detected per sample for the various tumour types tested (Fig 7A), as well as the distribution of mutations across the genes on the panel (Fig 7B). Among the successfully sequenced samples (97.4%, i.e., 342/351), at least one mutation was detected in 86.5% (296/342) of the samples, while 48.2% (165/342) of the samples had two or more mutations identified. In keeping with published studies, there were frequent mutations of *BRAF* and *NRAS* in melanoma; *BRAF*, *EGFR*, *KRAS*, and *STK11* in NSCLC; *APC*, *BRAF*, *KRAS*, and *PIK3CA* in CRC; *KIT* and *PDGFR3A* in GIST; and *CTNNB1* in other tumours (desmoid fibromatosis). *TP53* was the most frequently mutated gene across the cohort, with mutations in all tumour types.

The mutation rates detected with the panel were similar for many genes to those found in publicly available WES/WGS studies (S4 Fig) despite different portions of the genome being examined. A Fisher’s exact test was applied to these data for each tumour type on a per-gene basis, and the resulting *p*-values were corrected for multiplicity using a Bonferroni–Hochberg procedure [20] (S1 Data). This analysis demonstrated no statistically significant difference in the rates of mutation observed using the two different sequencing approaches for the majority of genes across all tumour types (only *p-*values < 0.05 were considered to be statistically significant).

Genes where a statistically significant difference in mutation rate was observed were *APC* (*p =* 9 × 10^−7^) in CRC, *STK11* (*p =* 0.008) in NSCLC, and *CDKN2A* (*p =* 0.02), *ERBB4* (*p =* 9 × 10^−4^), *FLT3* (*p =* 0.02), and *KDR* (*p =* 0.01) in melanoma. This finding suggests that although the panel covers only small amounts of the genome, it is very well targeted to the most frequently mutated regions: those genes where significantly more mutations were seen using WES/WGS compared to the panel have variants distributed across the gene rather than targeted on a few codons. This means that, despite its limited scope compared to WES/WGS, the panel is likely to capture most mutations in these genes, indicating its potential utility.

Full details of the mutations detected in the prospective cohort are given in S2 Data.

### Changes in patient management following cancer panel testing

Standard genetic testing of tumours is usually limited to situations where variant information will influence a specific intervention. In order to justify the cost of more extensive mutation analysis, it is necessary to demonstrate clinical utility, e.g., by analysing treatment decisions and patient outcomes based on data in electronic patient records. Given that the prescription of targeted therapies in the UK is regulated by NICE, evidence regarding clinical utility beyond NICE-approved indications is anecdotal.

Fig 7C demonstrates that the panel provided additional mutation information not detected by the cobas technology for the three “most actionable” genes: 34.2% (13/38) of *BRAF* mutations in melanoma, 19.0% (4/19) of *EGFR* mutations in NSCLC, and 11.1% (4/36) of *KRAS* mutations in CRC (only samples tested using both the panel and the cobas assay were included in this comparison). These mostly pertained to codons or variants outside the scope of the cobas assay, some of which are actionable. However, four additional *BRAF* V600E mutations in melanoma specimens were identified using the panel that should have been detected by the cobas (two failures and two false negatives). In contrast, cases where standard diagnostic assays provided additional information are entirely accounted for by panel sequencing failures.

Table 2 lists variants in the prospective patient cohort that did change, or could have changed, patient management given local availability of targeted therapeutics (assuming clinical criteria were met). In melanoma, guidance from NICE permits vemurafenib use for *BRAF* V600 mutated tumours, meaning one patient with a V600R mutation received the drug and others with V600G/M and V600K mutations were eligible. One patient with melanoma with a *KIT* V560D mutation entered a clinical trial of the TKI nilotinib, while another received the multi-targeted receptor TKI pazopanib after *NRAS* mutation status was determined (clinical trial entry required only knowledge of *NRAS* mutation status, not the detection of a mutation). NICE guidance regarding eligibility for vemurafenib in *BRAF* V600 mutated melanoma stipulates that patients have metastatic disease, but not all patients tested met this criterion, accounting for some of the discrepancy between actionable mutations and subsequent changes to patient management.

**Table 2:** Potentially actionable mutations detected in the prospective cohort using the cancer panel.

Tumour site (no. of specimens)^a^	Gene (no. of mutated samples)^b^	Identity of actioned / locally actionable mutation^c^	No. of samples with actioned / locally actionable mutation	No. of samples where actioned / locally actionable mutation only detected using Cancer Panel	No. patients receiving treatment based on mutation	No. patients treated where mutation information only available using Cancer Panel	Treatment Details	Supporting evidence
**Melanoma (107)**	*BRAF* (41)	V600E	30	4	10	2	8 patients received vemurafenib (further 20 patients eligible for vemurafenib based on mutation status)	Chapman *et al*. [23]
** **							2 patients received pan-RAF kinase inhibitor in clinical trial	Middleton *et al*. [24]
** **		V600G/V600M	1	1	0	0	Eligible for vemurafenib based on mutation status	Lang *et al*. [25]
** **		V600K	1	0	0	0	Eligible for vemurafenib based on mutation status	McArthur et al. [26]
** **		V600R	1	1	1	1	1 patient received vemurafenib	Klein *et al*. [27]
** **	*NRAS* (38)	Various^d^	1	1	1	1	1 patient received pazopanib in clinical trial	Dayer *et al*. [28]
** **			12^e^	12^e^	0	0	*NRAS* mutation status determined as part of screening process for clinical trial	
** **	*KIT* (4)	V560D	1	1	1	1	1 patient received nilotinib in clinical trial	Cho *et al*. [29]
**Non-small cell lung carcinoma (105)**	*EGFR* (21)	L858R	10	0	8	0	8 patients received erlotinib (further 2 patients eligible for *EGFR* inhibitor based on mutation status)	Rosell *et al*. [30]
** **		Exon 19 deletion	4	0	2	0	1 patient received erlotinib , 1 patient received gefitinib (further 2 patients eligible for *EGFR* inhibitor based on mutation status)	Zhou *et al*. [31]
** **		Exon 19 deletion/T790M	1	0	0	0	Likely to be resistant to erlotinib /gefitinib despite activating exon 19 mutation	Fukuoka *et al*. [32]
** **		E709K/G719C	1	0	0	0	Eligible for erlotinib/gefitinib based on mutation status	Pao *et al*. [33]
** **		G719A	1	0	0	0	Eligible for erlotinib/gefitinib based on mutation status	Chen *et al*. [34]
** **		M600T	1	1	1	1	1 patient received erlotinib	Han *et al*. [35]
** **		S720C	1	1	1	1	1 patient received erlotinib	Greulich *et al*. [36]
** **		V742I	1	1	1	1	1 patient received erlotinib	Avizienyte *et al*. [37]
** **		L861Q	1	1	1	1	1 patient received erlotinib	Watanabe *et al*. [38]
**Colorectal carcinoma (87)**	*KRAS* (37)	Activating mutations exon 2/3/4 (35 samples)	51 (total number of samples without activating KRAS or NRAS mutations)	51	5	5	5 patients lacking activating *KRAS* or *NRAS* mutations received cetuximab (further 46 patients eligible for anti-*EGFR* monoclonal antibody based on mutation status)	Van Cutsern *et al*. [39]
** **	*NRAS* (2)	Activating mutation exon 2/3 (2 samples)						Bokemeyer *et al*. [40]
** **								Stintzing *et al*. [41]
**GIST (14)**	*KIT* (5)	Exon 9 (0 samples)	13 (total number of samples without KIT exon 9 or PDGFRA D842V mutations)	13	6	6	6 patients lacking *KIT* exon 9 and *PDGFRA* D842V mutations received standard dose imatinib (remaining 7 patients lacking these mutations did not fit clinical criteria for treatment with a tyrosine kinase inhibitor i.e. low risk disease)	Heinrich *et al*. [42]
** **	*PDGFRA* (2)	D842V (1 samples)						Heinrich *et al*. [43]
**Breast (4)**	*PIK3CA* (1)	Various^d^	1	1	1	1	1 patient received BLY179 in clinical trial	Ciruelos *et al*. [19]
** **			3^f^	3^f^	0	0	*PIK3CA* mutation status determined as part of screening process for clinical trial	
**Ovarian Carcinoma; mucinous subtype (1)**	*KRAS* (0)	Activating mutations exon 2/3/4	1 (total number of samples without activating KRAS or NRAS mutations)	1	1	1	1 patient lacking activating *KRAS* or *NRAS* mutations received cetuximab	Sato *et al*. [44]
	*NRAS* (0)	Activating mutation exon 2/3						
			Total = 122^g^	Total = 78^g^	Total = 40	Total = 22		

^a^No. of samples refers to those successfully sequenced for each tumour type within the prospective cohort.

^b^No. of mutated samples includes samples with both actionable and non-actionable mutations in the relevant gene (see S2 Data for full mutation details).

^c^Locally actionable means there is a targeted therapy recommended by NICE Guidelines for the treatment of tumours of that histology harbouring that mutation (see S7 Table for a summary of NICE guidelines), or locally accessible clinical trial.

^d^Patient samples underwent Cancer Panel testing as part of the screening process for clinical trials of targeted therapeutics for melanoma and breast carcinoma to determine their mutation status for *NRAS* and *PIK3CA* respectively. In neither trial was it necessary for a patient’s tumour to have a mutation of the relevant gene for entry, rather the mutation status needed to be known.

^e^In total 13 patients with melanoma underwent screening for clinical trial entry, one of whom received pazopanib within a trial.

^f^In total 4 patients with breast carcinoma underwent screening for clinical trial entry, one of whom received BLY179 within a trial.

^g^Total excludes 15 patients who underwent screening for clinical trial entry (see ^e^ and ^f^) but did not enter a trial

NICE guidance requires only the presence of activating *EGFR* mutations [17,21] for NSCLC patients to be eligible for EGFR inhibitors, meaning four patients with unusual *EGFR* mutations (M600T, S720C, V742I, and L861Q) received erlotinib as a result of the panel. Evidence for the efficacy of *EGFR* inhibition in these mutations is scant due to their low frequency. There is some evidence that L861Q is an activating mutation although in vitro studies suggest it is sensitive to WZ-4002 (irreversible second-generation EGFR inhibitor) rather than erlotinib [22].

During this study, eligibility for anti-EGFR monoclonal antibody therapy (cetuximab or panitumumab) in CRC evolved from requiring wild-type *KRAS* to requiring wild-type *RAS*, necessitating mutation testing of *NRAS* in addition to *KRAS*. The only method of assessing *NRAS* status within our laboratory was the panel, and 51/88 (58.0%) patients with CRC were found to have tumours with wild-type *RAS*. The audit of subsequent clinical action revealed that 5/51 (9.8%) of these patients received anti-EGFR therapy (cetuximab). Further examination revealed that many of the 46 patients who on the basis of *RAS* mutation status would have been eligible for the treatment did not meet the clinical criteria (i.e., did not have metastatic disease; see S7 Table). Among CRC patients with mutated *RAS* tumours (37/88; 42.0%), four tumours would have been classed as wild-type *RAS* using conventional diagnostics: two had *NRAS* codon 61 mutations and two had *KRAS* codon 146 mutations (outside the scope of the cobas assay).

The panel was also the only mutation assay available for GIST patients at the time of analysis: although use of TKIs is dictated by clinical factors (moderate/high-risk or metastatic disease), certain mutations cause tumour resistance or require a higher TKI dose [45]. Six GIST patients received standard dose imatinib due to the absence of *KIT* exon 9 (requires higher dose) or *PDGFRA* D842V mutations (confers imatinib resistance). Four breast cancer patients had their *PIK3CA* mutation status determined, with one receiving BLY719 (PI3K inhibitor) in a clinical trial as a result, while a patient with a wild-type *RAS* mucinous ovarian carcinoma received cetuximab.

In total, 122/351 prospective cohort patients (34.8%) had a mutation for which there was either a NICE-approved targeted therapy or locally available clinical trial of a targeted therapy, 40 (32.8%) of whom received a targeted therapy. Fifty-five percent (22/40) of these patients received a targeted treatment only as a result of novel information from the panel. The additional 13 patients who received targeted therapies were NSCLC patients who received EGFR inhibitors second line in the absence of a previously detected activating *EGFR* mutation and patients with a variety of solid tumours who received targeted inhibitors in the context of a clinical trial where demonstration of a particular mutation was unnecessary.

A concern with testing progressively larger quantities of the tumour genome is that multiple potentially actionable mutations may be detected with conflicting recommended actions. Owing to the targeted nature of this panel centred around well-characterised hotspots and the paucity of targeted agents available in the UK, variant interpretation in this study was not impacted by this possibility. Where more than one mutation was identified in an actionable gene, there was either a clear hierarchy of action—e.g., NSCLC specimen G150739T had both an exon 19 deletion and p.T790M mutation in *EGFR*, meaning the patient would not respond to first generation EGFR TKIs but rather may benefit from a third generation drug [46]—or the same action was appropriate for both mutations, e.g., melanoma specimen G151372L had *BRAF* p.V600M and p.V600G mutations, both of which are likely to benefit from a BRAF inhibitor [25,47].

In order to investigate what the potential impact on clinical management of the panel might be in the future, we analysed the mutation data from the NSCLC and melanoma samples in the prospective cohort for theoretically actionable mutations as described by Meador et al. [48] (S5 Fig). A mutation was classified as theoretically actionable if there was peer-reviewed data at any level (from in vitro cell line to phase III randomised controlled trial [RCT] data) that indicated efficacy of an available treatment (predominantly targeted inhibitors). Assuming unrestricted access to these targeted therapies, 39.0% (41/105 successfully sequenced) of NSCLC and 66.4% (71/107 successfully sequenced) of melanoma patients had a potentially actionable mutation, in contrast to 20.0% (21/105) and 32.7% (35/107) of NSCLC and melanoma patients, respectively, who had a locally actionable mutation. This suggests that, in the future, far more of the variant information generated will lead to clinical management changes.

### Cost analysis

As shown in Table 3, the total cost for testing 46 genes using the panel was £339 (US$449) per sample (patient). This is compared to the cost of mutation testing with the single gene approach (cobas): £71 (US$94) for *BRAF*, £104 (US$138) for *EGFR*, and £141(US$187) for *KRAS*. Table 4 shows the cost by resource category for the different tests and for the combinations of these tests for different malignancies. For all tests, most costs are attributed to consumables, followed by staff and overheads. For example, the consumable cost is £185 per sample for the panel and between £34 and £93 for the cobas, depending on the gene tested.

**Table 3:** Test cost results.

Potential Testing Pathways	Cost per Sample in British Pounds (US Dollars)^a^
**Next generation sequencing**	
Cancer panel (Ion AmpliSeq)	£339 ($449)
**Single gene testing**	
cobas *BRAF*	£71 ($94)
cobas *EGFR*	£104 ($138)
cobas *KRAS*	£141 ($187)
cobas *PIK3CA*	£239 ($316)
**Test combinations**	
cobas *EGFR* and cancer panel	£422 ($559)
cobas *BRAF* and *KRAS*	£192 ($254)
cobas *BRAF*, *EGFR*, *KRAS*, and *PIK3CA*	£495 ($655)
cobas *BRAF*, *NRAS*, *KRAS*, and *PIK3CA*	£477 ($632)

Total cost per sample derived by summing the cost per stage and includes error costs, overheads, and miscellaneous costs such as staff training and staff turnover of 10% per year. These figures exclude the costs of value added tax.

^a^Costs converted into US dollars using XE Currency Converter (15 July 2016).

**Table 4:** Total cost per sample and type of malignancy by resource category.

Cost Item	Type of Test					Combination of Single Gene Tests for Different Malignancies		
	Ion AmpliSeq Panel	cobas *BRAF*	cobas *EGFR*	cobas *KRAS*	*KIT* (Sanger Sequencing)	NSCLC (*BRAF*, *EGFR*, *KRAS*)	Melanoma (*BRAF*, *NRAS*, *KIT*)	Colorectal Carcinoma (*KRAS*, *NRAS*)
**Equipment**	£18 (5%)	£0 (0%)	£0 (0%)	£0 (0%)	£0 (0%)	£1 (0%)	£1 (0%)	£0 (0%)
**Consumables**	£185 (55%)	£34 (48%)	£62 (59%)	£93 (66%)	£83 (60%)	£177 (64%)	£159 (57%)	£134 (65%)
**Staff**	£48 (14%)	£23 (33%)	£23 (22%)	£23 (16%)	£30 (21%)	£48 (17%)	£65 (23%)	£35 (17%)
**Miscellaneous^a^**	£31 (9%)	£2 (2%)	£2 (2%)	£2 (1%)	£2 (2%)	£5 (2%)	£5 (2%)	£3 (2%)
**Overheads**	£56 (17%)	£12 (17%)	£17 (17%)	£23 (17%)	£28 (17%)	£46 (17%)	£50 (18%)	£35 (17%)
**Total test cost**	£339 (100%)	£71 (100%)	£104 (100%)	£141 (100%)	£138 (100%)	£276 (100%)	£280 (100%)	£208 (100%)
**Total test cost in US dollars**	$449	$94	$138	$187	$183	$366	$371	$276

Data are given as cost in British pounds (percent of total cost), unless otherwise indicated.

^a^Miscellaneous costs for the Ion AmpliSeq panel are for Ion Reporter software, Ion Personal Genome Machine and Ion OneTouch maintenance contract, Ion AmpliSeq library preparation training, bioinformatics Ion software and data analysis starter package, staff training time for Personal Genome Machine System and AmpliSeq at manufacturer site, staff time for knowledge transfer of Personal Genome Machine training and bioinformatics in the laboratory, and staff turnover (10% per year). Miscellaneous costs for the cobas are for staff training time for the cobas at manufacturer site, staff time for knowledge transfer in the laboratory, and staff turnover (10% per year).

NSCLC, non-small-cell lung carcinoma.

## Discussion

We have validated and clinically implemented an NGS assay that detects relevant mutations across mutational hotspots of 46 genes from minimal quantities of FFPE-derived highly fragmented tumour DNA, allowing routine testing of multiple genes from small biopsies. Its performance has been validated across a variety of tumour types for single nucleotide variants and indels and has been shown to have an enhanced sensitivity compared with conventional diagnostic techniques. Treatment data revealed that surprisingly few patients (~40%) received any pharmacological treatment, targeted or otherwise, confounding the fact that for most patients the indication for mutation analysis should be to inform whether treatment should be conventional chemotherapy or targeted agents. Examination of individual cases showed that, in contravention of operational policy, many samples were not from metastatic or unresectable malignancies. Whilst early testing in the diagnostic pathway may be desirable, many targeted therapies are indicated only for metastatic or unresectable disease, such as erlotinib in NSCLC. Additionally, differences in side effects between conventional chemotherapy and targeted agents are such that some elderly patients may be deemed suitable for the latter but not the former, meaning that if no actionable mutation is identified there is no appropriate treatment.

Over a third of the patients in our prospective cohort (122/351) had a locally actionable mutation, with 40 patients receiving a targeted therapy, of which 22 received this therapy only because of tumour testing with the panel (three patients were able to access a clinical trial, 12 patients had access to NICE-sanctioned therapies, six patients had changes to their management in line with European Societal Guidelines, and one patient was able to access a therapy via local funding arrangements). Whilst these numbers are modest, they reflect the limited availability of targeted therapies approved by NICE. For example, significant numbers of patients within the prospective cohort could potentially have benefited from targeted therapies when data from just 7/46 genes were considered (*EGFR*, *KRAS*, *NRAS*, *BRAF*, *KIT*, *PDGFRA*, *PIK3CA*). Although not all these patients required treatment at this stage, others were unable to access drugs due to lack of either licensing for their specific mutation and tumour type or NICE approval, reflecting a lack of prospective phase III RCT data.

The panel provides flexibility to rapidly introduce new testing as novel therapies are licensed or eligibility criteria are updated, as was demonstrated with the introduction of *NRAS* testing in our cohort. The panel also enables patients’ eligibility for novel agents in clinical trials to be assessed, which provides a significant contribution to available treatment options.

It is important to note that the panel testing also highlights patients who should not receive treatment due to the presence of confounding activating mutations, in particular, CRC patients with activating mutations in *KRAS* or *NRAS*, for whom treatment with anti-EGFR monoclonal antibody is not indicated, and GIST patients with activating mutations in *KIT* or *PDGFRA*, who are not eligible for imatinib. This accounted for 38/351 patients (10.8%) in the prospective cohort (37 CRC patients with activating *KRAS* or *NRAS* mutations and one GIST patient with a *PDGFRA* D842V mutation) and is also an important outcome of tumour testing.

The panel described here enables parallel testing of multiple genes, in contrast to conventional diagnostic techniques. However, it must be noted that the panel covers hotspot mutations only, not full gene sequencing, and in its current format does not allow testing for larger copy number variants such as *HER2* gene amplification in breast cancer [49].

Furthermore, it should be recognised that although RCTs increasingly use mutation status in treatment stratification decisions, e.g., the Medical Research Council FOCUS4 trial [50], for rare mutations in frequently mutated genes, combinations of mutations, and any mutations in rarely mutated genes, it is unlikely that phase III RCTs will ever recruit sufficient patients to determine the most appropriate treatment option, particularly with ever-increasing numbers of inhibitors.

The alternative is to develop and join up worldwide repositories of genotype–phenotype data so all anecdotal experiences can be collated and statistically mined for commonality. For this approach to be informative, more molecularly directed access to novel treatment modalities of proven efficacy is required. In addition, clinical drug development has to systematically include genomic characterisation of patient samples to detect differential responses due to mutation signatures. An example of this is the National Lung Matrix trial, currently recruiting in the UK, which consists of parallel, multi-centre, single-arm phase II trials, each arm testing an experimental targeted drug in a population stratified by multiple prespecified target biomarkers employing a Bayesian adaptive design [51].

In terms of the affordability of NGS technologies for healthcare payers, Cancer Research UK has set several requirements for tumour profiling tests. One of these requirements is that the cost for such tests must be less than £300 [52]. Our cost analysis shows that the panel cost is only slightly higher than this, at £339 (US$449) per sample, and that for certain gene test combinations, such as testing for *BRAF*, *EGFR*, *KRAS*, and *PIK3CA* (Table 3), sequential single gene testing is actually more expensive than testing several genes at the same time using the panel (£27 for NSCLC and £32 for melanoma). In the context of other costing studies in this area, estimates show that there is considerable variation across studies [53]. For example, Gallego et al. reported a cost of £1,703 (US$2,700) for using a cancer panel in the diagnosis of CRC and polyposis syndromes [54], while Yorczyk et al. estimated the average cost for a single-tier hereditary 25-gene panel test using the MyRisk platform at £2,581 (US$4,099) per person [55]. Ghemlas et al. reported that the cost of NGS was £297 (US$470) per patient for genetic testing of inherited bone marrow failure [56]. There is also variation in what these studies include in the cost analysis, with some including only the costs of consumables. However, a common theme is that most of the cost estimates are substantially higher than the Cancer Research UK £300 target.

Further, our results suggest that, depending on the combination of genes tested, the panel can be less expensive than single gene testing. For example, if testing for melanoma is done using a combination of three single gene tests for *BRAF*, *NRAS*, and *KIT*, this costs less than the panel. However, in other contexts, if *KRAS* and *PIK3CA* or *EGFR* and *PIK3CA* were tested, then testing for only two genes would be more expensive than the cancer panel.

This study has a number of potential limitations, some of which are common to most NGS studies, and others of which are specific to our study. First, correct identification of variants requires access to high-quality tumour material; as with other studies, we used FFPE biopsies. Although sequencing was usually successful using this NGS assay, the formalin fixation process itself can cause alterations in the sequence due to deamination of cytosine to uracil [57]. Equally, detection of all the relevant variants in a tumour assumes a representative biopsy, which may not be the case given the phenomenon of clonal heterogeneity [58]. Second, as with most sequencing studies, assessment of the pathogenicity of variants whose clinical significance is not understood is challenging, and in the absence of biological data, many in silico variant effect prediction algorithms have limitations.

Specific study limitations relate to our use of real word data: submission of cases for sequencing did not always follow the suggested algorithms (see S1 Fig) and therefore, regardless of the variants detected, the patients were not eligible for targeted treatments on clinical grounds. Owing to some cases being submitted from other institutions, not all clinical information was available for all patients, reducing the overall power of the study. Finally, limited access to some targeted therapies within the UK NHS meant that even if there was a potential drug indicated for a variant in a particular tumour type, often this drug would not be funded for use. In terms of our cost analysis, due to the limited availability of single gene cost data, the comparison between the Ion AmpliSeq panel and cobas was based on only three genes, whereas the comparison should ideally be made on the cost of the nine genes that have known clinical value. At the same time, we believe that the use of real-life data in this setting was the most appropriate way to study the true clinical utility and cost-effectiveness of the panel in a given healthcare system.

### Conclusions

In conclusion, in the context of the UK NHS, we validated and translated a cancer panel that reports clinically actionable results back to clinicians. This led to actionable mutations being identified in 122/351 (34.8%) patients in our cohort and allowed 15.3% (22/144) of those receiving pharmacological treatment to have access to targeted therapies not indicated using conventional single gene testing, thereby demonstrating the ability of the panel to impact clinical management. Our results also show that providing a mutation analysis service using a cancer panel for cancer diagnostics and treatment would provide value for money for the NHS, because if several genes are tested individually, which is often the case, then a one-stop test would require less resources (and time) and be less expensive than sequential gene testing. Using cancer panels for molecular testing will help molecular diagnostic laboratories deal with the increased demand for genetic testing and rapidly and reliably detect relevant mutations, even for a limited amount of tumour tissue, at a relatively low cost. Currently, only 9/46 genes on the panel are recognised to have clinical utility in the UK system, but as additional genes are clinically validated as targets, greater potential of NGS technologies could be realised.

## Supporting information

S1 SQUIRE Checklist (PDF)

S1 STARD Checklist (DOCX)

S1 Data Statistical analysis of comparative gene mutation frequencies for cancer panel and whole exome sequencing/whole genome sequencing studies. (XLSX)

S2 Data All mutations detected in the prospective cohort. (XLSX)

S1 Fig Genetic testing algorithms for samples. (TIFF)

S2 Fig Technical validation using retrospective cohort 1. (TIFF)

S3 Fig Concordance between mutation results obtained using the cancer panel and other diagnostic techniques. (TIFF)

S4 Fig Comparison of cancer panel gene mutation frequencies with whole exome sequencing/whole genome sequencing studies. (TIFF)

S5 Fig Potentially clinically actionable mutations. (TIFF)

S1 Table Assays used for validation by cohort. (DOCX)

S2 Table Design of the cancer panel. (DOCX)

S3 Table Sequencing reagents and reaction conditions. (DOCX)

S4 Table Mutation concordance between cancer panel and standard diagnostic techniques. (DOCX)

S5 Table Validation of mutations outside genes/regions covered by standard diagnostic techniques. (DOCX)

S6 Table Validation of mutations detected using cancer panel in retrospective cohort 2. (DOCX)

S7 Table Summary of National Institute for Health and Care Excellence guidelines for targeted cancer therapeutics. (DOCX)

S1 Text Technical validation of the cancer panel. (DOCX)

S2 Text Costing questionnaires. (DOCX)

## Abbreviations

CRC: colorectal carcinoma
FFPE: formalin-fixed paraffin-embedded
GIST: gastrointestinal stromal tumour
NGS: next generation sequencing
NHS: National Health Service
NICE: National Institute for Health and Care Excellence
NSCLC: non-small-cell lung carcinoma
RCT: randomised controlled trial
TKI: tyrosine kinase inhibitor
WES: whole exome sequencing
WGS: whole genome sequencing
