# Supplementary figures and images for: Clinical applicability and cost of a 46-gene panel for genomic analysis of solid tumours: Retrospective validation and prospective audit in the UK National Health Service
Source: PLoS Med. 2017 Feb 14;14(2):e1002230. doi: 10.1371/journal.pmed.1002230 (PMC5308858; doi:10.1371/journal.pmed.1002230)

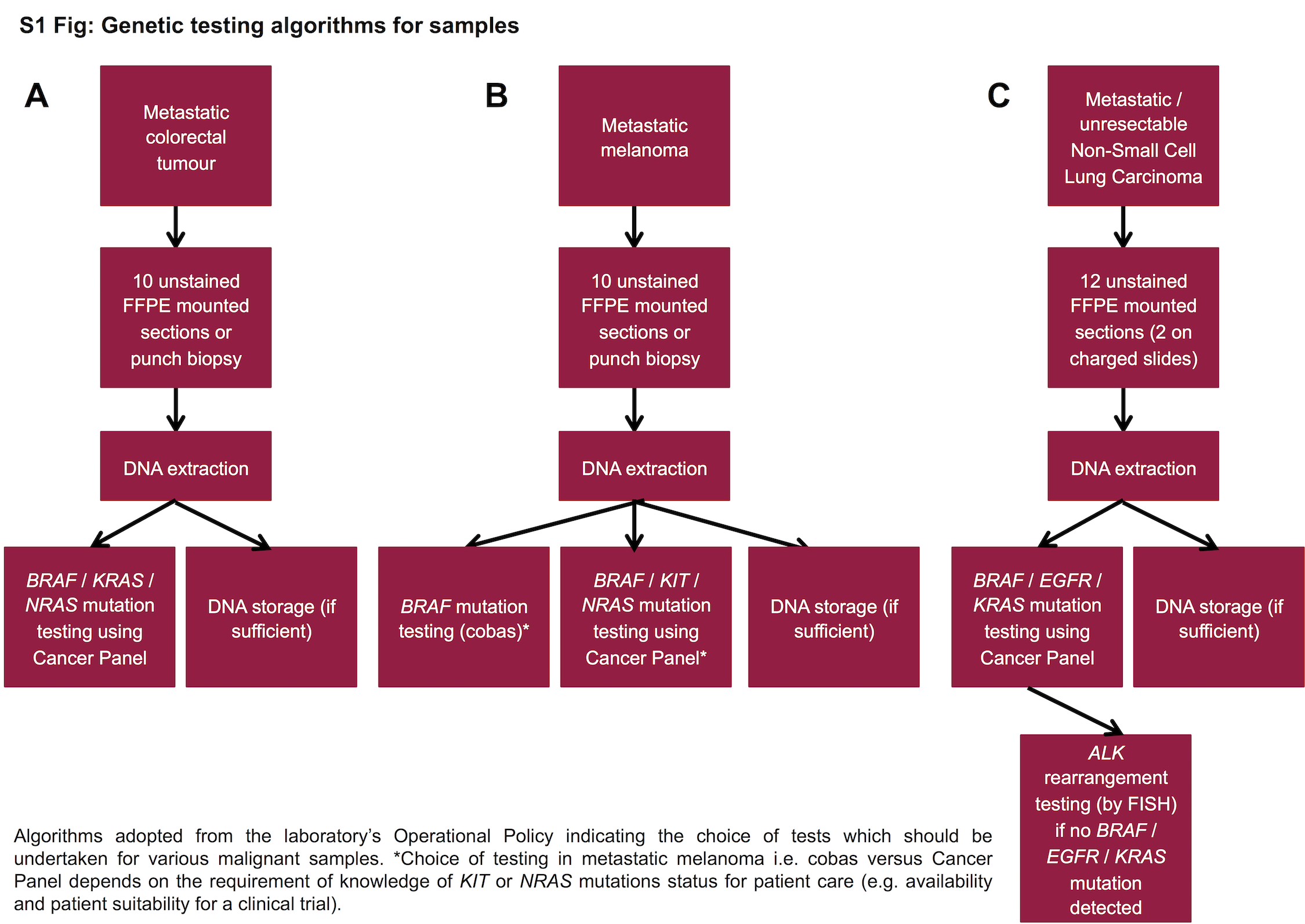

Supplement: S1 Fig — (TIFF) [file pmed.1002230.s005.tiff]

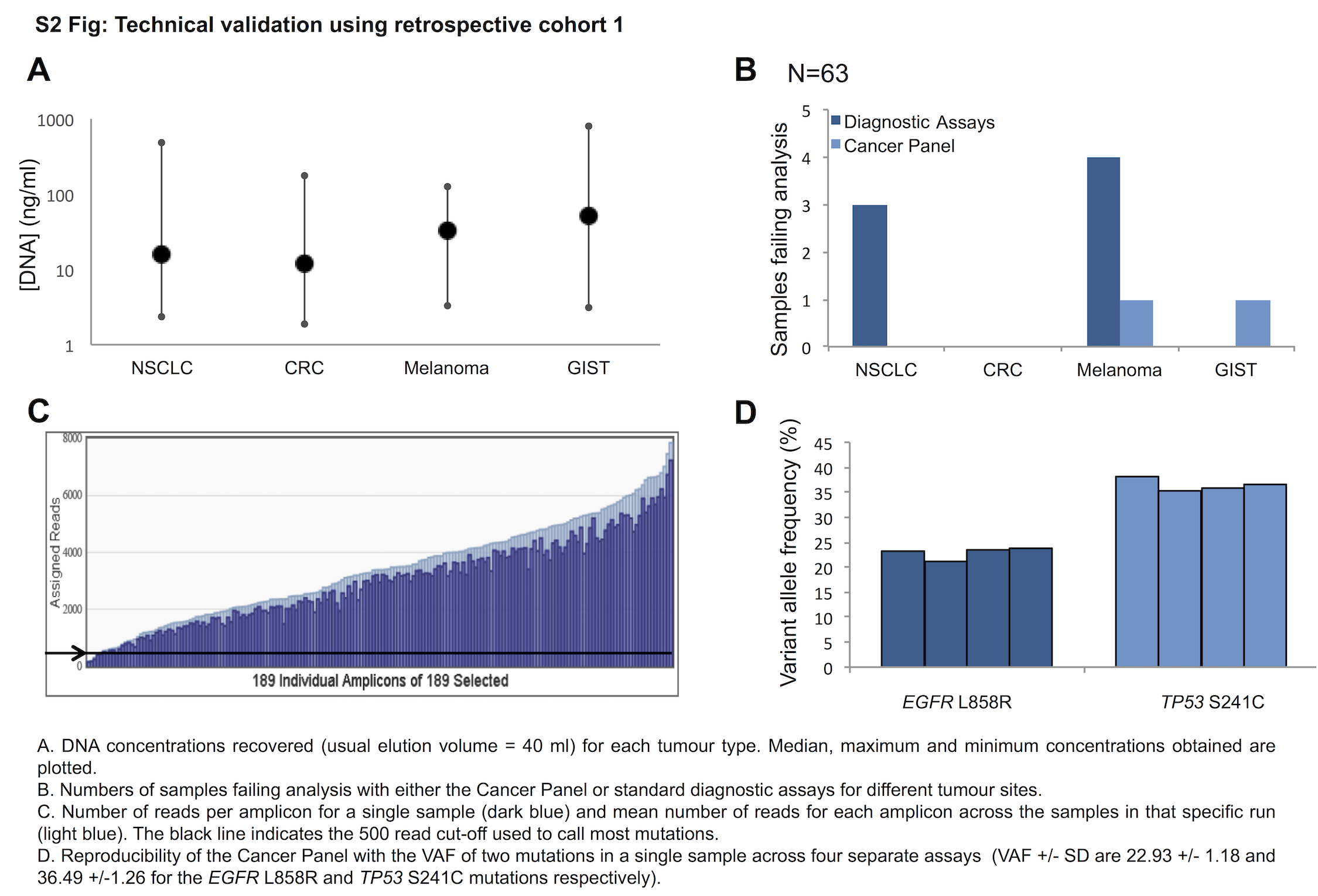

Supplement: S2 Fig — (TIFF) [file pmed.1002230.s006.tiff]

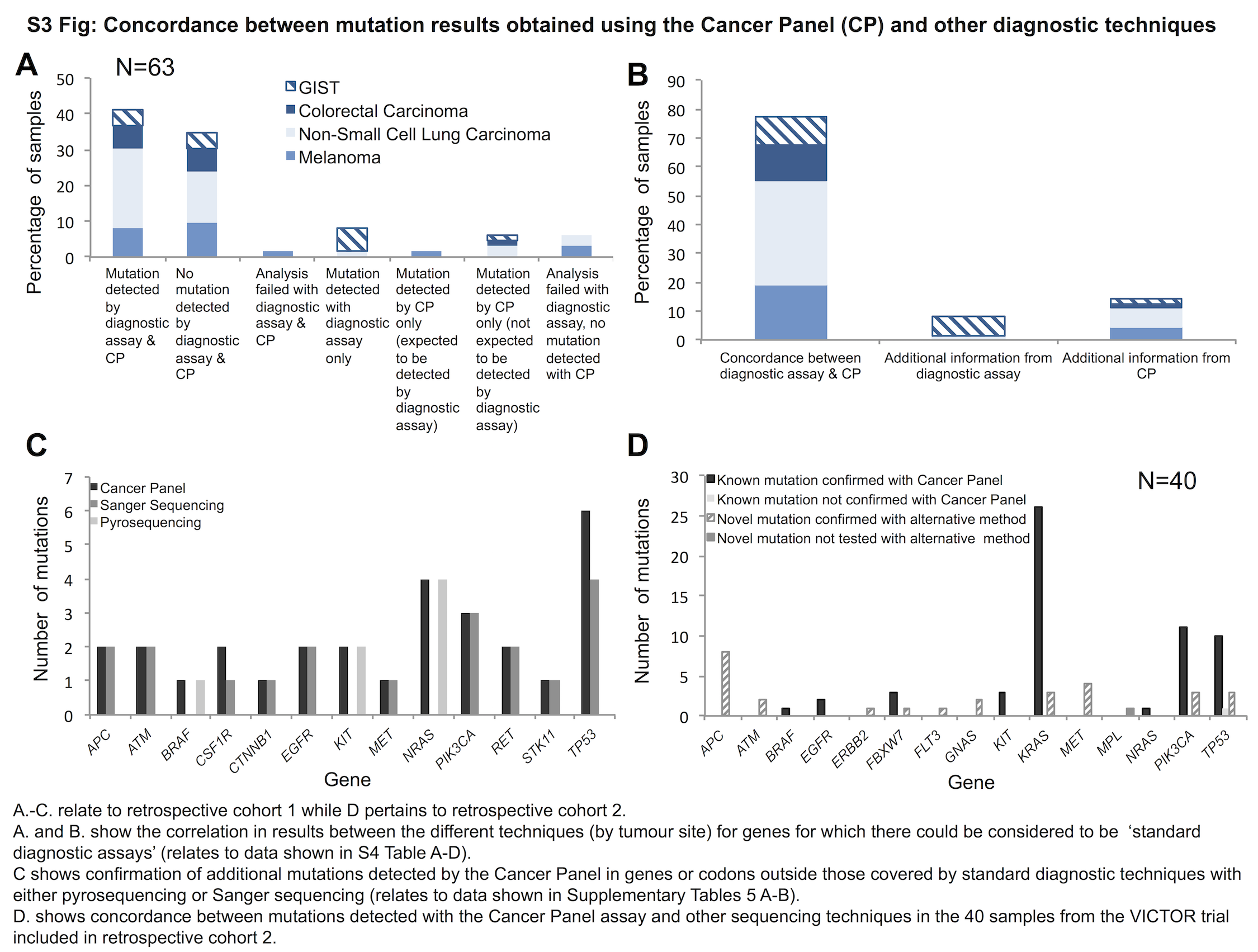

Supplement: S3 Fig — (TIFF) [file pmed.1002230.s007.tiff]

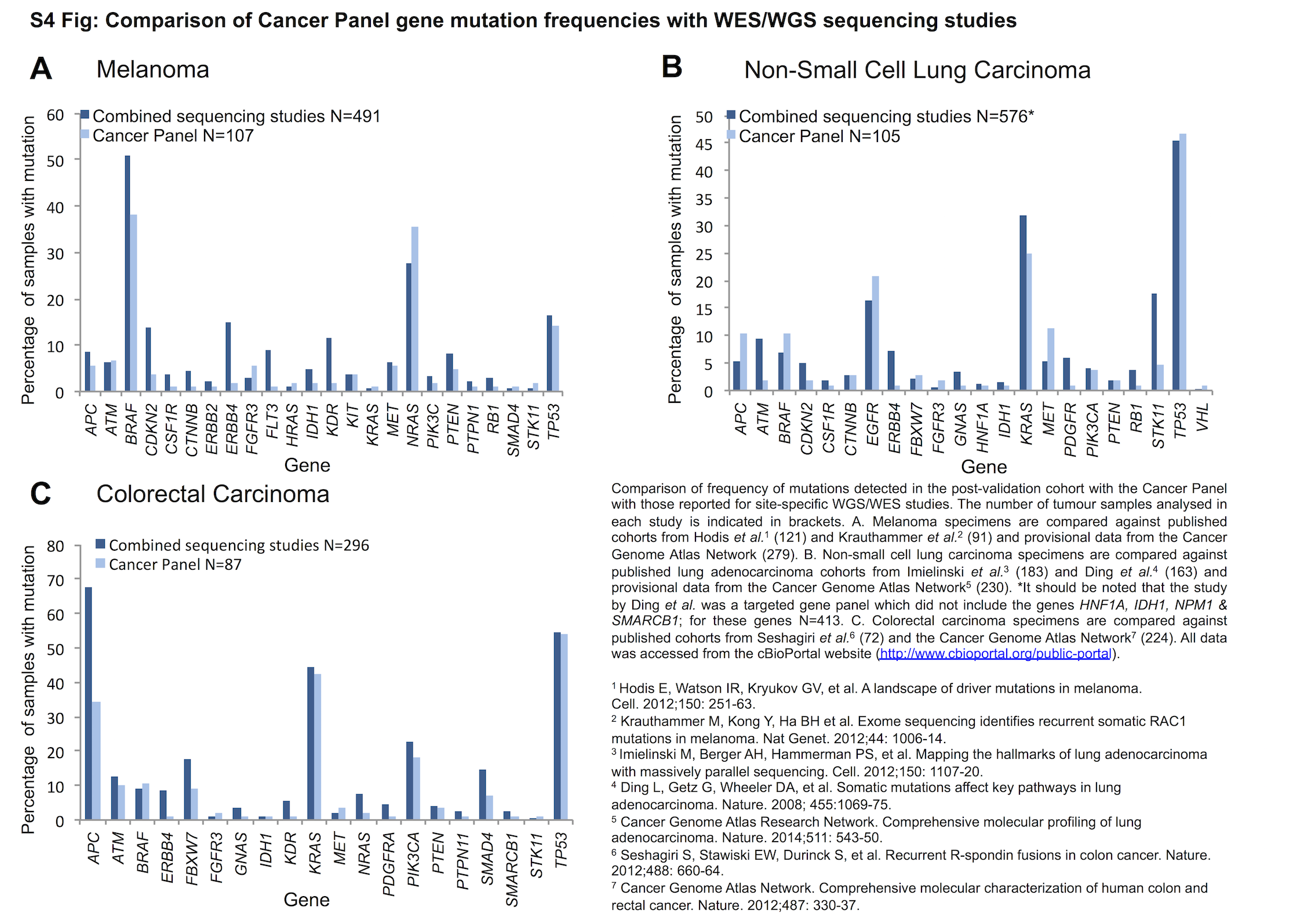

Supplement: S4 Fig — (TIFF) [file pmed.1002230.s008.tiff]

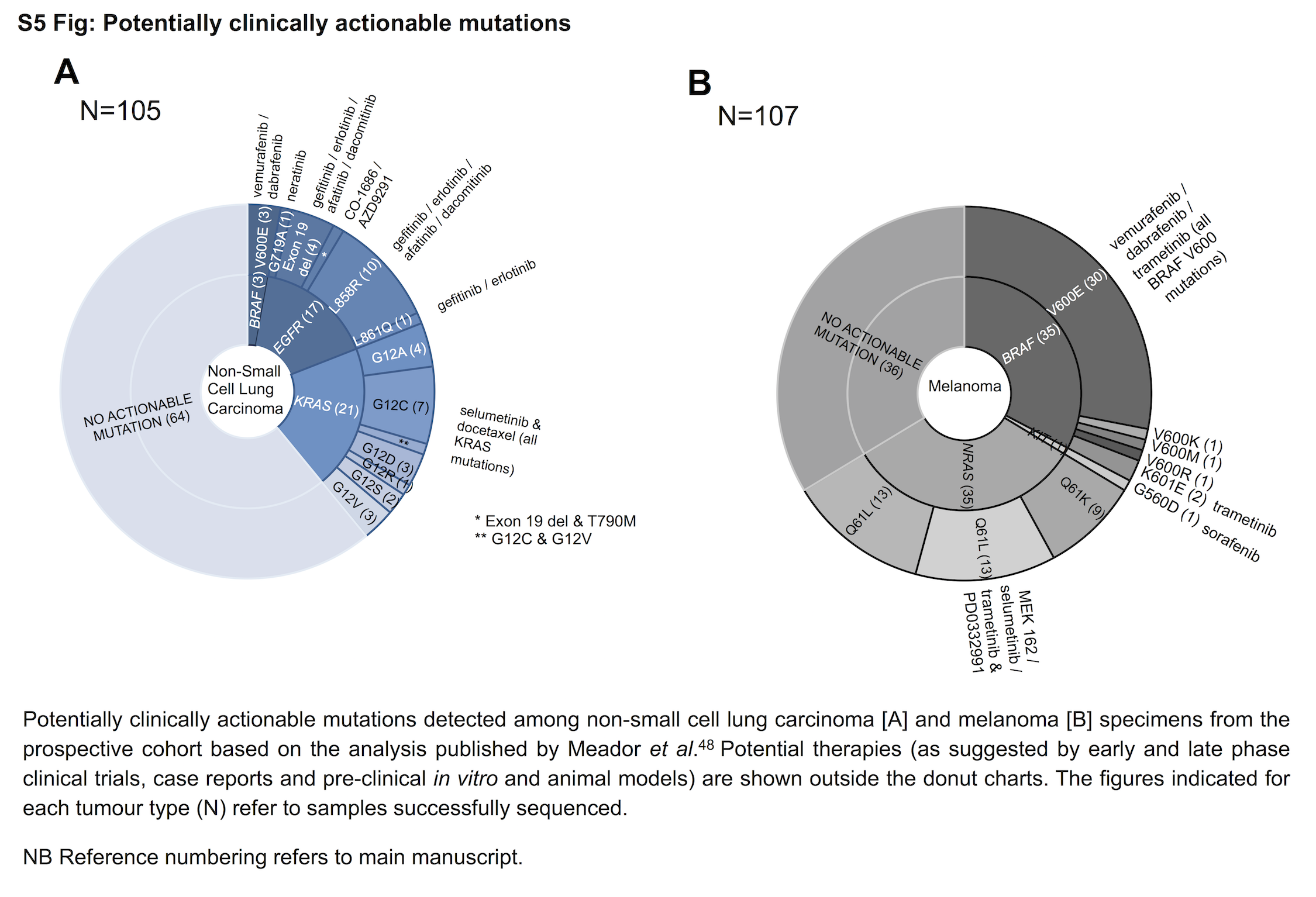

Supplement: S5 Fig — (TIFF) [file pmed.1002230.s009.tiff]
