# Supplementary material for: Clinical applicability and cost of a 46-gene panel for genomic analysis of solid tumours: Retrospective validation and prospective audit in the UK National Health Service
Source: PLoS Med. 2017 Feb 14;14(2):e1002230. doi: 10.1371/journal.pmed.1002230 (PMC5308858; doi:10.1371/journal.pmed.1002230)
Supplement: S1 Table — (DOCX) [file pmed.1002230.s010.docx]

**S1 Table: Assays used for validation by cohort**

| **Cohort** | **Diagnostic / Confirmatory Testing** | **Tumour Type** | **Technique** | **Gene(s)** |
| --- | --- | --- | --- | --- |
| Retrospective: Cohort 1 | Diagnostic  Confirmatory | CRC  GIST  Melanoma  NSCLC  CRC  GIST  Melanoma  NSCLC | Pyrosequencing  Sanger sequencing  Pyrosequencing  Fragment Analysis  Pyrosequencing  Pyrosequencing  Sanger sequencing  Pyrosequencing  Pyrosequencing  Sanger sequencing  Sanger sequencing | *BRAF / KRAS*  *KIT*  *BRAF / KRAS*  *EGFR*  *EGFR / KRAS*  *BRAF*  *APC / CSF1R / CTNNB1 / MET / PIK3CA / TP53*  *KIT*  *KIT / NRAS*  *ATM / CSF1R / RET / STK11 / TP53*  *ATM / EGFR / PIK3CA / TP53* |
| Retrospective: Cohort 2 | Diagnostic  Confirmatory | CRC  GIST  NSCLC  CRC | Sanger sequencing  Sanger sequencing  Fragment analysis  Alternative NGS assay  Sanger sequencing | *BRAF / FBXW7 / KRAS / NRAS / PIK3CA / TP53*  *KIT*  *EGFR*  *APC / ERBB2 / FBXW7 / FLT3 / KRAS / PIK3CA / TP53*  *ATM / GNAS / MET* |
| Prospective | Diagnostic | CRC  Melanoma  NSCLC | cobas  cobas  cobas | *KRAS*  *BRAF*  *BRAF / EGFR / KRAS* |

Assay type and scope used with the different patient cohorts in diagnostic (i.e. testing prior to the Panel) or confirmatory capacities (i.e. testing performed to confirm novel mutations detected using the Panel).
