# Supplementary material for: Clinical applicability and cost of a 46-gene panel for genomic analysis of solid tumours: Retrospective validation and prospective audit in the UK National Health Service
Source: PLoS Med. 2017 Feb 14;14(2):e1002230. doi: 10.1371/journal.pmed.1002230 (PMC5308858; doi:10.1371/journal.pmed.1002230)
Supplement: S2 Table — (DOCX) [file pmed.1002230.s011.docx]

**S2 Table: Design of the Cancer Panel**

| **Gene** | **Exons** | **Gene** | **Exons** |
| --- | --- | --- | --- |
| ***ABL1*** | 4, 5, 6, 7 | ***JAK3*** | 13, 16 |
| ***AKT1*** | 3 | ***KDR*** | 6, 7, 11, 19, 21, 26, 27, 30 |
| ***ALK*** | 23, 25 | ***KIT*** | 2, 9, 10, 11, 13, 14, 15, 17, 18 |
| ***APC*** | 14 | ***KRAS*** | 2, 3, 4 |
| ***ATM*** | 8, 9, 12, 26, 34, 35, 36, 39, 50, 54, 55, 56, 59, 61, 63 | ***MET*** | 2, 14, 16, 19 |
| ***BRAF*** | 11, 15 | ***MLH1*** | 12 |
| ***CDH1*** | 3, 8, 9 | ***MPL*** | 10 |
| ***CDKN2A*** | 2 | ***NOTCH1*** | 26, 27 |
| ***CSF1R*** | 7, 22 | ***NPM1*** | 11 |
| ***CTNNB1*** | 3 | ***NRAS*** | 2, 3, 4 |
| ***EGFR*** | 3, 7, 15, 18, 19, 20, 21 | ***PDGFRA*** | 12, 14, 15, 18 |
| ***ERBB2*** | 19, 20, 21 | ***PIK3CA*** | 2, 5, 8, 10, 14, 21 |
| ***ERBB4*** | 3, 4, 6, 7, 8, 9, 15, 23 | ***PTEN*** | 1, 3, 6, 7, 8 |
| ***FBXW7*** | 5, 8, 9, 10 | ***PTPN11*** | 3, 13 |
| ***FGFR1*** | 4, 7 | ***RB1*** | 4, 6, 11, 17, 18, 20, 21, 22 |
| ***FGFR2*** | 7, 9, 12 | ***RET*** | 10, 11, 13, 15, 16 |
| ***FGFR3*** | 7, 9, 14, 16, 18 | ***SMAD4*** | 3, 5, 6, 8, 9, 10, 11, 12 |
| ***FLT3*** | 11, 14, 16, 20 | ***SMARCB1*** | 2, 4, 5, 9 |
| ***GNAS*** | 8 | ***SMO*** | 3, 5, 6, 9, 11 |
| ***HNF1A*** | 3, 4 | ***SRC*** | 14 |
| ***HRAS*** | 2, 3 | ***STK11*** | 4, 6, 8, 22 |
| ***IDH1*** | 4 | ***TP53*** | 2, 4, 5, 6, 7, 8, 10 |
| ***JAK2*** | 14 | ***VHL*** | 1, 2, 3 |

Genes and their constituent exons (partially) covered by the Panel. Due to the targeted nature of the assay, many exons are not covered in their entirety with coverage centred around the mutation hotspots.
