# Supplementary material for: Clinical applicability and cost of a 46-gene panel for genomic analysis of solid tumours: Retrospective validation and prospective audit in the UK National Health Service
Source: PLoS Med. 2017 Feb 14;14(2):e1002230. doi: 10.1371/journal.pmed.1002230 (PMC5308858; doi:10.1371/journal.pmed.1002230)
Supplement: S3 Table — (DOCX) [file pmed.1002230.s012.docx]

**S3 Table: Sequencing Reagents and Reaction Conditions**

**A**

| Gene | Region / Variant | Primer sequence (5’-3’) | Primer concentration (nmol/L) | DNA volume (μl) | Qiagen mastermix volume (μl) | Q solution volume (μl) | Reaction volume (μl) | PCR Programme |
| --- | --- | --- | --- | --- | --- | --- | --- | --- |
| *APC* | Q1349X | Fwd: TCAGACGACACAGGAAGCAG  Rev: GCTGAGATCAGCCAAATTCAGTT | 200 | 1 | 12.5 | 2.5 | 25 | 1 |
| *APC* | R1432X | Fwd: GAATCAGCCAGGCACAAAGC  Rev: TGAGTGGGGTCTCCTGAACA | 200 | 1 | 12.5 | 2.5 | 25 | 1 |
| *ATM* | F858L | Fwd: TGTGCCCAGCCTGATTAGGTA  Rev: TTGGCTCTCTCCAGGTTCGT | 200 | 1 | 12.5 | 2.5 | 25 | 1 |
| *ATM* | R3008S | Fwd: TGTGCCCAGCCTGATTAGGTA  Rev: TTGGCTCTCTCCAGGTTCGT | 200 | 1 | 12.5 | 2.5 | 25 | 1 |
| *CSF1R* | A299T | Fwd: GCATTAGCAAGCTTGGGCTC  Rev: CCAGATGCTTGTGTGTTCTGC | 200 | 1 | 12.5 | 2.5 | 25 | 1 |
| *CSF1R* | E317G | Fwd: GTTGTCGGGCTGTGTAGACG  Rev: GGGACTGGATCAATGGGTGG | 200 | 1 | 12.5 | 2.5 | 25 | 1 |
| *CTNNB1* | T41A | Fwd: TGAGCTAACCCTGGCTATCATT  Rev: CCCTGTTCCCACTCATACAGG | 200 | 1 | 12.5 | 2.5 | 25 | 1 |
| *EGFR* | G719A | Fwd: CTGAGGTGACCCTTGTCTCTG  Rev: TGTGCCAGGGACCTTACCTT | 200 | 1 | 12.5 | 2.5 | 25 | 1 |
| *EGFR* | V769M | Fwd: ATTCATGCGTCTTCACCTGGA  Rev: TGTCTTTGTGTTCCCGGACAT | 200 | 1 | 12.5 | 2.5 | 25 | 1 |
| *KIT* | Exon 11 | Fwd: CCAGAGTGCTCTAATGACTG  Rev: ACCCAAAAAGGTGACATGGA | 200 | 1 | 12.5 | 0 | 25 | 2 |
| *MET* | N375S | Fwd: GATCGATCTGCCATGTGTGC  Rev: AGTGGGGAACTGATGTGACTT | 200 | 1 | 12.5 | 2.5 | 25 | 1 |
| *PIK3CA* | E542K & E545K | Fwd: TGTGAATCCAGAGGGGAAAAAT  Rev: GCTGAGATCAGCCAAATTCAGTT | 200 | 1 | 12.5 | 2.5 | 25 | 1 |
| *RET* | R770Q & D771N | Fwd: TTATGTGAACATCATTCAAGGCG  Rev: GCAGGACTGTCAAGCAGAGAA | 200 | 1 | 12.5 | 2.5 | 25 | 1 |
| *STK11* | F354L | Fwd: CCACTGCTTCTGGGCGTTTG  Rev: GGGCAGAAGCTGTCCTTGTT | 200 | 1 | 12.5 | 2.5 | 25 | 1 |
| *TP53* | R26P | Fwd: TGCCGTCTTCCAGTTGCT  Rev: CAGTGAGGAATCAGAGGCCTG | 200 | 1 | 12.5 | 2.5 | 25 | 1 |
| *TP53* | R64X & R81X | Fwd: CAGGCCTCTGATTCCTCACTGATTGCTC  Rev: CTCACCTGGAGGGCCACTGACAA | 200 | 1 | 12.5 | 2.5 | 25 | 1 |
| *TP53* | G113D | Fwd: CAAGGCGCACTGGCCTCATCT  Rev: CAGGCCACTGTGCAGGGT | 200 | 1 | 12.5 | 2.5 | 25 | 1 |
| *TP53* | R141H, R141L & P146S | Fwd: GGTAGGACCTGATTTCCTTACT  Rev: AGGCATAACTGCACCCTTGG | 200 | 1 | 12.5 | 2.5 | 25 | 1 |

Primers and reagents used in PCR amplification reactions for Sanger sequencing.

| Gene  **B** | Region / Variant | Primer sequence (5’-3’) | Sequencing primer | Primer concentration (nmol/L) | DNA volume (μl) | Qiagen mastermix volume (μl) | Q solution volume (μl) | Reaction volume (μl) | PCR Programme |
| --- | --- | --- | --- | --- | --- | --- | --- | --- | --- |
| *BRAF* | Codon 600 & F595L | Fwd: CTTTACTTACTACACCTCAGA  Rev: CTCAATTCTTACCATCCACAA | TGATTTTGGTCTAGCTACA (600)  TCACAGTAAAAATAGGTGA (595) | 300 | 1 | 12.5 | 0 | 25 | 3 |
| *EGFR* | L858 | Fwd: ATTCGGATGCAGAGCTTCTT  Rev: CCTCCTTCTGCATGGTATTC | AGATCACAGATTTTGGG | 200 | 1 | 12.5 | 0 | 25 | 4 |
| *KIT* | D816V & N822K | Fwd: TCATGGTCGGATCACAAAGAT  Rev: CAGGACTGTCAAGCAGAGAATGG | TGATTTTGGTCTAGCCAG | 400 | 1 | 12.5 | 0 | 25 | 5 |
| *KRAS* | Codon 12/13 | Fwd: GGCCTGCTGAAAATGACTGA  Rev: AGAATGGTCCTGCACCAGTAATA | CTTGTGGTAGTTGGAG | 200 | 1 | 12.5 | 0 | 25 | 4 |
| *KRAS* | Codon 61 | Fwd: TGTTTCTCCCTTCTCAGGATTC  Rev: AAGAAAGCCCTCCCCAGTC | GGATATTCTCGACACAGC | 200 | 1 | 12.5 | 0 | 25 | 4 |
| *NRAS* | Codon 61 | As per Qiagen Kit | As per Qiagen Kit | As per Qiagen Kit | As per Qiagen Kit | As per Qiagen Kit | As per Qiagen Kit | As per Qiagen Kit | As per Qiagen Kit |

Primers and reagents used in PCR amplification reactions for pyrosequencing.

**C**

| Gene | Region / Variant | Primer sequence (5’-3’) | Primer concentration (nmol/L) | DNA volume (μl) | Qiagen mastermix volume (μl) | Q solution volume (μl) | Reaction volume (μl) | PCR Programme |
| --- | --- | --- | --- | --- | --- | --- | --- | --- |
| *EGFR* | Exon 19 | Fwd: GCATGTGGCACCATCTCAC  Rev: FAM - GAGGTTCAGAGCCATGGAC | 100 | 1 | 12.5 | 5 | 25 | 4 |

Primers and reagents used in PCR amplification for fragment analysis.

**D**

| PCR programme | Initialisation | | Cycle | | | | | | Number of cycles | Final elongation | |
| --- | --- | --- | --- | --- | --- | --- | --- | --- | --- | --- | --- |
|  | Temp | Time | Denaturation | | Annealing | | Elongation | |  | Temp | Time |
|  |  |  | Temp | Time | Temp | Time | Temp | Time |  |  |  |
| 1 | 95 ^o^C | 10 min | 95 ^o^C | 1 min | 62 ^o^C | 45 s | 72 ^o^C | 45 s | 35 | 72 ^o^C | 10 min |
| 2 | 97 ^o^C | 15 min | 97 ^o^C | 30 s | 58 ^o^C | 90 s | 72 ^o^C | 2 min | 50 | 72 ^o^C | 10 min |
| 3 | 95 ^o^C | 10 min | 95 ^o^C | 15 s | 55 ^o^C | 1 min | 72 ^o^C | 1 min | 40 | 72 ^o^C | 10 min |
| 4 | 95 ^o^C | 10 min | 95 ^o^C | 15 s | 55 ^o^C | 1 min | 72 ^o^C | 1 min | 35 | 72 ^o^C | 10 min |
| 5 | 95 ^o^C | 15 min | 95 ^o^C | 30 s | 65 ^o^C | 90 s | 72 ^o^C | 45 s | 35 | 72 ^o^C | 10 min |

PCR programme conditions used for Sanger sequencing, pyrosequencing and fragment analysis amplification reactions.
