# Supplementary material for: Clinical applicability and cost of a 46-gene panel for genomic analysis of solid tumours: Retrospective validation and prospective audit in the UK National Health Service
Source: PLoS Med. 2017 Feb 14;14(2):e1002230. doi: 10.1371/journal.pmed.1002230 (PMC5308858; doi:10.1371/journal.pmed.1002230)
Supplement: S4 Table — (DOCX) [file pmed.1002230.s013.docx]

**S4 Table: Mutation concordance between Cancer Panel and standard diagnostic techniques**

**A**

| **Sample ID** | **Tumour specimen** | **Result using standard diagnostic techniques** | **Result using Cancer Panel** |
| --- | --- | --- | --- |
| G125266L | NSCLC | *EGFR* L858R | *EGFR* L858R |
| G123786W | NSCLC | *EGFR* L858R | *EGFR* L858R |
| G121669T | NSCLC | *EGFR* Exon 19 Del | *EGFR* Exon 19 Del |
| G126337Q | NSCLC | *KRAS* G12D | *KRAS* G12D |
| G126727R | NSCLC | *KRAS* G13C | *KRAS* G13C |
| G126728P | NSCLC | Negative | Negative |
| G126741S | NSCLC | Negative | Negative |
| G126756V | NSCLC | Negative | Negative |
| G126888Q | NSCLC | Negative | Negative |
| G126889V | NSCLC | Negative | Negative |
| G126884D | NSCLC | *KRAS* G12C | *KRAS* G12C |
| G126886G | NSCLC | *KRAS* G12C | *KRAS* G12C |
| G126887K | NSCLC | Negative | Negative |
| G127018F | NSCLC | *EGFR* L858R | *EGFR* L858R |
| G126832V | NSCLC | *EGFR* L858R | *EGFR* L858R |
| G126933N | NSCLC | Negative | Negative |
| G126936R | NSCLC | *EGFR* Exon 19 Del | Negative |
| G127149W | NSCLC | *EGFR* L858R | *EGFR* L858R |
| G127153K | NSCLC | Failed | Negative |
| G127154Q | NSCLC | Failed | *KRAS* A146T |
| G127842A | NSCLC | Negative | Negative |
| G127551G | NSCLC | *EGFR* L858R | *EGFR* L858R |
| G127488P | NSCLC | *KRAS* G12V | *KRAS* G12V |
| G127861A | NSCLC | *EGFR* Exon 19 Del | *EGFR* Exon 19 Del |
| G125878F | NSCLC | *EGFR* Exon 19 Del | *EGFR* Exon 19 Del |
| G128225N | NSCLC | Negative | *EGFR* G719A; V769M |
| G128273G | NSCLC | Failed | Negative |
| G128284H | NSCLC | Negative | Negative |

Concordance in mutation results obtained for non-small cell lung carcinoma samples (N=28) in retrospective cohort 1.

**B**

| **Sample ID** | **Tumour specimen** | **Result using standard diagnostic techniques** | **Result using Cancer Panel** |
| --- | --- | --- | --- |
| G125841G | CRC | Negative | Negative |
| G126466D | CRC | Negative | Negative |
| G127148B | CRC | Negative | *BRAF* F595L, *KRAS* A146T |
| G127150D | CRC | *KRAS* G12V | *KRAS* G12V |
| G127151F | CRC | Negative | Negative |
| G127837K | CRC | Negative | Negative |
| G127489L | CRC | *KRAS* Q61H | *KRAS* Q61H |
| G128125Y | CRC | *KRAS* G13D | *KRAS* G13D |
| G128224T | CRC | *BRAF* V600E | *BRAF* V600E |

Concordance in mutation results obtained for colorectal carcinoma samples (N=9) in retrospective cohort 1.

**C**

| **Sample ID** | **Tumour specimen** | **Result using standard diagnostic techniques** | **Result using Cancer Panel** |
| --- | --- | --- | --- |
| G126332W | Melanoma | *BRAF* V600E | *BRAF* V600E |
| G126730B | Melanoma | Negative | Negative |
| G126731W | Melanoma | Failed | Negative |
| G126893S | Melanoma | Failed | Negative |
| G126983Q | Melanoma | *BRAF* V600E | *BRAF* V600E |
| G126935H | Melanoma | Negative | Negative |
| G127157X | Melanoma | Failed | *BRAF* V600E |
| G127844T | Melanoma | Negative | Negative |
| G127845N | Melanoma | Failed | Failed |
| G127851B | Melanoma | *KRAS* G12A | *KRAS* G12A |
| G127390J | Melanoma | Negative | Negative |
| G127394L | Melanoma | *BRAF* V600E | *BRAF* V600E |
| G127928W | Melanoma | Negative | Negative |
| G128149N | Melanoma | *BRAF* V600K | *BRAF* V600K |
| G127671A | Melanoma | negative | negative |

Concordance in mutation results obtained for melanoma samples (N=15) in retrospective cohort 1.

**D**

| **Sample ID** | **Tumour specimen** | **Result using standard diagnostic techniques** | **Result using Cancer Panel** |
| --- | --- | --- | --- |
| G125149H | GIST | *KIT* 12bp deletion | Negative |
| G126194H | GIST | Negative | Negative |
| G125689G | GIST | *KIT* 12bp deletion | Negative |
| G125790A | GIST | Negative | Negative |
| G126466D | GIST | *KIT* 12bp deletion | Failed |
| G127271X | GIST | Negative | *KIT* N822K |
| G127395B | GIST | Negative | Negative |
| G129815V | GIST | *KIT* 6bp deletion | *KIT* 6bp deletion |
| G130141A | GIST | *KIT* 6bp deletion | Negative |
| G130016L | GIST | *KIT* 6bp deletion | *KIT* 6bp deletion |
| G130015P | GIST | *KIT* 6bp deletion | *KIT* 6bp deletion |

Concordance in mutation results obtained for GIST samples (N=11) in retrospective cohort 1.
