# Supplementary material for: Clinical applicability and cost of a 46-gene panel for genomic analysis of solid tumours: Retrospective validation and prospective audit in the UK National Health Service
Source: PLoS Med. 2017 Feb 14;14(2):e1002230. doi: 10.1371/journal.pmed.1002230 (PMC5308858; doi:10.1371/journal.pmed.1002230)
Supplement: S5 Table — (DOCX) [file pmed.1002230.s014.docx]

**S5 Table: Validation of mutations outside genes / regions covered by standard diagnostic techniques**

**A**

| **Sample ID** | **Tumour specimen** | **Mutation detected using Cancer Panel** | **VAF using Cancer Panel (%)** | **Mutation confirmed using pyrosequencing** | **VAF using pyrosequencing (%)** |
| --- | --- | --- | --- | --- | --- |
| G127148B | CRC | *BRAF* F595L | 36.15 | YES | 26 |
| G129948V | Melanoma | *KIT* D816V | 46.26 | YES | 47 |
| G127271X | GIST | *KIT* N822K | 54.28 | YES | 52 |
| G126731W | Melanoma | *NRAS* Q61K | 17.58 | YES | 14 |
| G127928W | Melanoma | *NRAS* Q61L | 30.55 | YES | 29 |
| G126730B | Melanoma | *NRAS* Q61R | 62.82 | YES | 58 |
| G127390J | Melanoma | *NRAS* Q61R | 56.97 | YES | 61 |

Mutations in genes / regions outside those covered by standard diagnostic techniques detected using the Panel in retrospective cohort 1 samples confirmed using pyrosequencing. VAFs for the two techniques are shown.

**B**

| **Sample ID** | **Tumour specimen** | **Mutation detected using Cancer Panel** | **VAF using Cancer Panel (%)** | **Mutation confirmed using Sanger sequencing** |
| --- | --- | --- | --- | --- |
| G127150D | CRC | *APC* Q1349X | 42.46 | YES |
| G127148B | CRC | *APC* R1432X | 39.57 | YES |
| G127928W | Melanoma | *ATM* F858L | 79.75 | YES |
| G126886G | NSCLC | *ATM* R3008S | 37.21 | YES |
| G127395B | CRC | *CSF1R* A299T | 49.87 | YES |
| G126332W | Melanoma | *CSF1R* E317G | 6.67 | NO |
| G126194H | CRC | *CTNNB1* T41A | 42.19 | YES |
| G128225N | NSCLC | *EGFR* G719A | 18.35 | YES |
| G128225N | NSCLC | *EGFR* V769M | 55.51 | YES |
| G127395B | CRC | *MET* N375S | 67.79 | YES |
| G127151F | CRC | *PIK3CA* E542K | 14.85 | YES |
| G126337Q | NSCLC | *PIK3CA* E542K | 11 | YES (weak) |
| G126337Q | NSCLC | *PIK3CA* E545K | 8.64 | YES (weak) |
| G126935H | Melanoma | *RET* R770Q | 40.19 | YES |
| G126935H | Melanoma | *RET* D771N | 44.35 | YES |
| G127928W | Melanoma | *STK11* F354L | 48.6 | YES |
| G127861A | NSCLC | *TP53* G113D | 39.16 | YES |
| G126730B | Melanoma | *TP53* P146S | 42.03 | YES |
| G128224T | CRC | *TP53* R141H | 45.97 | YES |
| G126756V | NSCLC | *TP53* R141L | 92 | YES |
| G126888Q | NSCLC | *TP53* R26P | 27.37 | YES |
| G126887K | NSCLC | *TP53* R64X | 18.2 | NO |
| G126731W | Melanoma | *TP53* R81X | 8.1 | NO |

Mutations in genes / regions outside those covered by standard diagnostic techniques detected using the Panel in retrospective cohort 1 samples confirmed using Sanger sequencing. VAFs for the Panel are shown.
