# Supplementary material for: Clinical applicability and cost of a 46-gene panel for genomic analysis of solid tumours: Retrospective validation and prospective audit in the UK National Health Service
Source: PLoS Med. 2017 Feb 14;14(2):e1002230. doi: 10.1371/journal.pmed.1002230 (PMC5308858; doi:10.1371/journal.pmed.1002230)
Supplement: S6 Table — (DOCX) [file pmed.1002230.s015.docx]

**S6 Table: Validation of mutations detected using Cancer Panel in retrospective cohort 2**

**A**

| **Sample** | **Mutation** | **VAF (%)** |
| --- | --- | --- |
| A01 | *FBXW7*:R465C | 32.89 |
| A01 | *KRAS*:G12V | 50.23 |
| A01 | *PIK3CA*:E545K | 44.44 |
| B01 | *KRAS*:G12C | 13.78 |
| C01 | *KRAS*:G12D | 33.2 |
| D01 | *TP53*:R248Q | 43.07 |
| E01 | *FBXW7*:R465C | 58.22 |
| E01 | *TP53*:R273H | 58.67 |
| F01 | *KRAS*:G12D | 37.19 |
| G01 | *KRAS*:G12D | 25.52 |
| G01 | *PIK3CA*:E545K | 14.68 |
| H01 | *BRAF*:V600E | 33.6 |
| B02 | *KRAS*:G13D | 31.36 |
| B02 | *PIK3CA*:E542K | 30.77 |
| C02 | *TP53*:R273H | 55.78 |
| D02 | *TP53*:R273L | 42.76 |
| E02 | *FBXW7*:R465C | 17 |
| E02 | *KRAS*:G12D | 19.46 |
| F02 | *KRAS*:G12D | 47.17 |
| G02 | *KRAS*:G12D | 25.03 |
| G02 | *TP53*:R306X | 35.47 |
| H02 | *KRAS*:G12V | 26.96 |
| A03 | *KRAS*:G12V | 21.39 |
| B03 | *KRAS*:G12D | 6.91 |
| C03 | *KRAS*:G12D | 51.75 |
| D03 | *KRAS*:G12C | 23.47 |
| D03 | *TP53*:R282W | 42.34 |
| E03 | *PIK3CA*:E545K | 9.63 |
| F03 | *TP53*:R306X | False Negative |
| G03 | *KRAS*:G12A | 27.29 |
| H03 | *KRAS*:G12V | 25.07 |
| A04 | *PIK3CA*:E545K | 15.07 |
| B04 | *KRAS*:G12D | 17.69 |
| B04 | *PIK3CA*:E542K | 12.62 |
| C04 | *KRAS*:G12D | 19.71 |
| C04 | *PIK3CA*:E545K | 19.56 |
| D04 | *KRAS*:G12C | 39.65 |
| E04 | *KRAS*:G12D | 23.33 |
| E04 | *TP53*:R248W | 38.31 |
| F04 | *KRAS*:G12V | 10.22 |
| G04 | *KRAS*:G13D | 39.56 |
| H04 | *NRAS*:Q61L | 36.29 |
| A05 | *PIK3CA*:E545K | 22.27 |
| B05 | *KRAS*:G12R | 27.32 |
| C05 | *KRAS*:G13D | 25.82 |
| C05 | *PIK3CA*:H1047R | 34.72 |
| C05 | *TP53*:R175H | 39.6 |
| D05 | *KRAS*:G12D | 48.09 |
| E05 | *TP53*:R273H | 50 |
| F05 | *KRAS*:G12V | 17.68 |
| F05 | *TP53*:R213X | 38.5 |
| G05 | *PIK3CA*:E545K | 14.65 |
| H05 | *PIK3CA*:E545K | 21.24 |

Mutations (SNVs) detected in retrospective cohort 2 samples using the Panel previously identified using Sanger sequencing (with the exception of *TP53*:R248W which was previously identified using Sanger sequencing but not confirmed with the Panel).

**B**

| **Sample** | **Mutation** | **VAF (%)** |
| --- | --- | --- |
| 8063D | *KIT* exon 11 28 bp deletion | N/A |
| 8965S | *EGFR* exon 19 12 bp deletion | 8 |
| 9496A | *KIT* exon 11 30 bp insertion | 100 |
| 0081Q | *KIT* exon 11 51 bp deletion | 100 |
| 0331X | *EGFR* exon 19 18 bp deletion | 47 |

Mutations (indels) detected in retrospective cohort 2 samples using the Panel previously identified with fragment analysis or Sanger sequencing.

**C**

| **Sample** | **Mutation** | **VAF (%)** | **Confirmation technique** |
| --- | --- | --- | --- |
| C01 | *APC*:R876X | 18.05 | Alternative NGS assay |
| A02 | *APC*:Y1376X | 19.38 | Alternative NGS assay |
| A02 | *ERBB2*:V777L | 77.85 | Alternative NGS assay |
| A02 | *TP53*:K132E | 48.35 | Alternative NGS assay |
| B02 | *ATM*:V410A | 54.71 | Sanger sequencing |
| B02 | *MET*:E168D | 53.55 | Sanger sequencing |
| C02 | *APC*:Q1291X | 53.4 | Alternative NGS assay |
| G02 | *APC*:R876X | 11.11 | Alternative NGS assay |
| G02 | *APC*:R1450X | 7.17 | Alternative NGS assay |
| *A03* | *PIK3CA:E542K* | *10.99* | *Alternative NGS assay* |
| B03 | *FLT3*:A680V | 6.64 | Alternative NGS assay |
| E03 | *KRAS*:G12V | 10.56 | Alternative NGS assay |
| *F03* | *KRAS:G13D* | *24.23* | *Alternative NGS assay* |
| B04 | *GNAS*:R201H | 11.29 | Sanger sequencing |
| C04 | *FBXW7*:R278X | 23.03 | Alternative NGS assay |
| *C04* | *TP53:R248W* | *25.59* | *Alternative NGS assay* |
| D04 | *APC*:Q1291X | 32.86 | Alternative NGS assay |
| E04 | *MET*:N375S | 41.4 | Sanger sequencing |
| G04 | *APC*:Y1376X | 73.67 | Alternative NGS assay |
| G04 | *GNAS*:R201H | 24.27 | Sanger sequencing |
| *G04* | *TP53:R248W* | *14.15* | *Alternative NGS assay* |
| *A05* | *KRAS:G12V* | *13.52* | *Alternative NGS assay* |
| *B05* | *PIK3CA:E542K* | *12.62* | *Alternative NGS assay* |
| D05 | *MET*:T1010I | 59.96 | Sanger sequencing |
| *D05* | *PIK3CA:H1047L* | *19.65* | *Alternative NGS assay* |
| E05 | *APC*:R876X | 45 | Alternative NGS assay |
| E05 | *MET*:T1010I | 49.2 | Sanger sequencing |
| G05 | *ATM*:F858L | 64.43 | Sanger sequencing |
| 9496A | *MPL*:R524C | 9 | Not tested |

Mutations detected in retrospective cohort 2 samples using the Panel, not previously identified with conventional testing. The variants shown in italics are those identified using the Panel which were not identified using Sanger sequencing despite being within the amplicons evaluated using this.
