# Supplementary material for: Clinical applicability and cost of a 46-gene panel for genomic analysis of solid tumours: Retrospective validation and prospective audit in the UK National Health Service
Source: PLoS Med. 2017 Feb 14;14(2):e1002230. doi: 10.1371/journal.pmed.1002230 (PMC5308858; doi:10.1371/journal.pmed.1002230)
Supplement: S7 Table — (DOCX) [file pmed.1002230.s016.docx]

**S7 Table: Summary of NICE Guidelines for targeted cancer therapeutics**

| **Tumour Site** | **Targeted Therapy** | **NICE Guidance** | **Recommendation** | **Further Information** |
| --- | --- | --- | --- | --- |
| Melanoma | Vemurafenib | TA269^[[1]](#endnote-1)^ | Vemurafenib is recommended as an option for treating *BRAF* V600 mutation-positive unresectable or metastatic melanoma |  |
| Non-small Cell Lung Carcinoma | Gefitinib  Erlotinib | TA192^[[2]](#endnote-2)^  TA258^[[3]](#endnote-3)^ | Gefitinib is recommended as an option for the first-line treatment of people with locally advanced or metastatic non-small-cell lung cancer (NSCLC) if they test positive for the epidermal growth factor receptor tyrosine kinase (*EGFR*-TK) mutation  Erlotinib is recommended as an option for the first-line treatment of people with locally advanced or metastatic non-small-cell lung cancer (NSCLC) if they test positive for the epidermal growth factor receptor tyrosine kinase (*EGFR*-TK) mutation | The UK marketing authorisation for gefitinib clarifies that the *EGFR*-TK mutation should be **activating** but no further definition of such mutations is given  The UK marketing authorisation for erlotinib clarifies that the *EGFR*-TK mutation should be **activating** but no further definition of such mutations is given |
| Colorectal Carcinoma | Cetuximab | TA176^[[4]](#endnote-4)^ | Cetuximab in combination with FOLFOX or FOLFIRI chemotherapy, within its licensed indication, is recommended for the first-line treatment of metastatic colorectal cancer only if the primary colorectal tumour has been resected or is potentially operable, the metastatic disease is confined to the liver and is unresectable and the patient is fit enough to undergo surgery to resect the primary colorectal tumour and to undergo liver surgery if the metastases become resectable after treatment with cetuximab | The UK marketing authorisation for cetuximab clarifies that the metastatic colorectal cancer should be *RAS* wild-type  Within the UK the permitted use of cetuximab is extended by the Cancer Drug Fund^[[5]](#endnote-5)^ recommendations to include:   - First-line treatment in combination with irinotecan-based chemotherapy or FOLFOX4 or FOLFOX6 or OxMdG in RAS wild-type metastatic colorectal carcinoma not meeting the specifications of NICE guidance TA176 - Third or fourth-line treatment as a single agent for *RAS* wild-type metastatic colorectal carcinoma providing ECOG Performance Status <2 |
| GIST | Imatinib | TA326^[[6]](#endnote-6)^ | Imatinib is recommended as an option as adjuvant treatment for up to 3 years for adults who are at high risk of relapse after surgery for KIT (CD117)-positive gastrointestinal stromal tumours | Details of patients at high risk of relapse and those with a tumour mutational profile likely to render them either insensitive or requiring higher doses of imatinib are contained within ESMO/European Sarcoma Network Working Group: [Gastrointestinal stromal tumors: ESMO Clinical Practice Guidelines for diagnosis, treatment and follow-up](http://www.ncbi.nlm.nih.gov/pubmed/22997454)^[[7]](#endnote-7)^ |

Summary of NICE Guidance permitting prescribing of targeted cancer therapeutics within the publically funded NHS in the UK.

1. National Institute for Health and Care Excellence: Vemurafenib for treating locally advanced or metastatic BRAF V600 mutation-positive malignant melanoma [TAG269]. London, National Institute for Health and Care Excellence, 2012 [↑](#endnote-ref-1)
2. National Institute for Health and Care Excellence: Gefitinib for the first-line treatment of locally advanced or metastatic non-small-cell lung cancer [TAG192]. London, National Institute for Health and Care Excellence, 2010 [↑](#endnote-ref-2)
3. National Institute for Health and Care Excellence: Erlotinib for the first-line treatment of locally advanced or metastatic EGFR-TK mutation-positive non-small-cell lung cancer [TA258]. London, National Institute for Health and Care Excellence, 2012 [↑](#endnote-ref-3)
4. National Institute for Health and Care Excellence: Cetuximab for the first-line treatment of metastatic colorectal cancer [TAG176]. London, National Institute for Health and Care Excellence, 2009 [↑](#endnote-ref-4)
5. The Cancer Drugs Fund. http://www.england.nhs.uk/ourwork/pe/cdf/ [↑](#endnote-ref-5)
6. National Institute for Health and Care Excellence: Imatinib for the adjuvant treatment of gastrointestinal stromal tumours (review of NICE technology appraisal guidance 196) [TA326]. London, National Institute for Health and Care Excellence, 2014 [↑](#endnote-ref-6)
7. ESMO/European Sarcoma Network Working Group: [Gastrointestinal stromal tumors: ESMO Clinical Practice Guidelines for diagnosis, treatment and follow-up.](http://www.ncbi.nlm.nih.gov/pubmed/22997454) Ann Oncol 25:S21-S26, 2014 (Suppl 3) [↑](#endnote-ref-7)
