# Supplementary material for: Clinical applicability and cost of a 46-gene panel for genomic analysis of solid tumours: Retrospective validation and prospective audit in the UK National Health Service
Source: PLoS Med. 2017 Feb 14;14(2):e1002230. doi: 10.1371/journal.pmed.1002230 (PMC5308858; doi:10.1371/journal.pmed.1002230)
Supplement: S1 Text — (DOCX) [file pmed.1002230.s017.docx]

**S1 Text: Technical validation of the Cancer Panel**

**Methods**

**Retrospective cohort details**

The retrospective cohort comprised cohort 1 (N=63) and cohort 2 (N=45) samples. The former were tested in tandem with standard diagnostic assays (see below and S1 Table) while the latter, was composed of 40 colorectal cancer (CRC) samples from the VICTOR study (Vioxx In Colorectal cancer Therapy: definition of Optimal Regime)^^[[1]](#endnote-1)^^ previously characterised for mutations within known hotspots of the *BRAF*, *FBXW7*, *KRAS*, *NRAS*, *PIK3CA* and *TP53* genes and 5 non-small cell lung cancer (NSCLC) and mesenchymal tumours with known indels in *EGFR* or *KIT* respectively. Novel mutations detected in cohort 2 were confirmed using either Sanger sequencing or a custom-designed 150 whole gene NGS panel (Thermo Fisher).

**Sample preparation**

Retrospective cohort 1 and prospective cohort samples underwent senior histopathological review and areas with maximum malignant cell density were marked on an H&E stained slide. Corresponding areas were macrodissected from five to ten 5 μm sections and DNA was extracted using the QIAamp DNA FFPE Tissue Kit (Qiagen) according to the manufacturer’s instructions. Alternatively a punch biopsy was taken from the tumour block from the area of maximum malignant cell density and DNA extracted using the QIAamp DNA FFPE Tissue Kit. DNA was extracted from retrospective cohort 2 as previously described.^[[2]](#endnote-2)^ DNA from all samples was quantified with a Qubit using the DNA High Sensitivity Kit (Invitrogen).

**Sanger sequencing**

Among retrospective cohort 1 samples, Sanger sequencing was used in a diagnostic capacity for detecting mutations in *KIT* exon 11 and in a confirmatory role for mutations in *APC*, *ATM*, *CSF1R*, *CTNNB1*, *EGFR*, *MET*, *PIK3CA*, *RET*, *STK11* and *TP53*. Primers, reagent concentrations and PCR conditions for the amplification reactions are listed in Tables 3A and 4. Bidirectional sequencing was performed on each sample using BigDye terminator chemistry and an ABI 3130 Genetic Analyser (both Thermo Fisher) and mutation analysis was performed using Mutation Surveyor (Softgenetics). Mutations in the retrospective cohort 2 were characterised using Sanger sequencing as previously described.^[[3]](#endnote-3)^

**Pyrosequencing**

Among retrospective cohort 1 samples pyrosequencing was used in a diagnostic capacity for *BRAF* codon 600, *EGFR* L858 and *KRAS* codons 12, 31 and 61 mutations and in a confirmatory role for mutations in *KIT*, *NRAS* and non-codon 600 variants in *BRAF*. Primers (designed in-house with the exception of those for *NRAS* codon 61 which were from the *therascreen* *NRAS* Pyro Kit [Qiagen]), reagent concentrations and PCR conditions for the amplification reactions are listed in Tables 3B and 4. A 20 μl aliquot of each PCR product underwent pyrosequencing using PyroMark Q24 (Qiagen) according to the manufacturer’s instructions and data were analysed with the machine’s in-built software.

**Fragment analysis**

Fragment analysis was used in a diagnostic capacity in the retrospective cohort for *EGFR* exon 19 deletions. Primers, reagent concentrations and PCR conditions for the amplification reaction are listed in Tables 3C and 4. The PCR product was diluted 1:50 in nuclease-free water and 1 μl was mixed with 8 μl Hi-Di formamide and 0.5 μl GeneScan 500 ROX Size Standard (both Thermo Fisher) before being denatured by heating to 95 ^o^C for 3 min. Fragment analysis was performed using an ABI 3130 Genetic Analyser and data were analysed using GeneMapper v4.1 software (Thermo Fisher).

**cobas**

Among the prospective cohort the majority of melanoma, NSCLC and CRC specimens were tested for *BRAF* V600, activating *EGFR* and *KRAS* codon 12/13 and 61 mutations respectively using the cobas platform (Roche) in a diagnostic capacity in tandem with analysis by the Cancer Panel (Panel). These assays, all based on real-time PCR, were performed as per the manufacturer’s instructions.

**NGS Data Analysis**

Initially NGS data analysis was performed using Torrent Suite software (TSS) version 2.2 with variants being called by the Torrent Suite Variant Caller (TVC) plugin using Ion AmpliSeq library type and Somatic variant frequency settings. The targeted regions and hotspots for this plugin were defined using the appropriate BED files for the AmpliSeq Cancer Panel (available on the Ion Community website; <http://ioncommunity.lifetechnologies.com>). Variants were filtered to remove those with a total coverage of <500x and a frequency of <4% with the exception of indels. The min-var-freq plugin parameter settings for the detection of indels was lowered from 19% to 1% after failure to detect *EGFR* exon 19 deletions that had been previously demonstrated amongst retrospective cohort 1 samples using fragment analysis. Remaining variants were inspected visually in IGV^[[4]](#endnote-4)^ (Broad Institute) for changes in read depth and false positives. Retrospective cohort 2 samples which underwent sequencing with the 150 gene panel to confirm novel mutations detected by the Panel underwent analysis using an Ion Reporter Tumour/Normal workflow with the 150 gene panel BED file.

Part way through the prospective cohort NGS sequence analysis moved to TSS version 3.4.2 and Ion Reporter 1.4. Data was automatically uploaded from TSS to Ion Reporter (IR) on the completion of a sequencing run and were aligned to hg19. Variants were called and annotated using a customised workflow: Quality control of amplicon performance was executed using output files from the TVC in conjunction with the Coverage Analysis plugin. As before variants with <500x coverage were removed as were variants with frequency of <4% or <8% depending on whether they were at the centre of a hotspot or a surrounding residue (e.g. *BRAF* V600E versus *BRAF* K601E respectively). This bioinformatics change was instigated to provide greater automation of the analysis pipeline enabling it to be executed within the laboratory by clinical scientists without the use of custom scripts.

Further filtering was performed to remove synonymous mutations, common polymorphisms (e.g. those included in dbSNP136^[[5]](#endnote-5)^ or the 1000 genomes project^[[6]](#endnote-6)^) and likely false positive variant calls (identified by high recurrence rates, absence on visual inspection and/or negative pyrosequencing or Sanger Sequencing; list available on request). Changes to either reagents (e.g. 316 to 318 chips) or analysis pipelines (e.g. TSS version 2.2 to TSS version 3.4.2 and IR version 1.4) were validated either *in silico* or by re-sequencing samples.

**Results**

**Technical assessment of the Cancer Panel using retrospective cohort 1**

The use of FFPE-derived DNA for sequencing applications can be challenging due to frequent low yields of poor quality DNA recovered after the fixation and paraffin-embedding processes. However, even the lowest DNA yields for each tumour type were adequate for analysis using the Panel ( S2 Fig A) and the assay failure rate across the tumour sites among these samples (N=63) was only 3.2% (compared with 11.1% for the same samples across different conventional diagnostic techniques, S2 Fig B). One of the two samples that failed Panel analysis was a melanoma sample that also failed conventional analysis, probably due to high levels of melanin known to interfere with PCR amplification.^[[7]](#endnote-7)^

The median number of bases per run for 316 and 318 chips was 172M (range 122-228M) and 528M (range 387-729M) respectively with >90% typically passing filter (Q20). S2 Fig C shows a representative example of coverage across the 189 amplicons analysed. Evaluation of mean amplicon coverage across each amplicon in each sample gave a median coverage depth of 2782x and typically five amplicons were excluded from analysis owing to coverage of <500x. Post-filtering inter-run variant allele frequency (VAF) variation was minimal (S2 Fig D).

**Comparison of concordance between mutations detected using the Cancer Panel and standard diagnostic techniques in retrospective cohort 1**

The 63 samples in retrospective cohort 1 (28 NSCLC, 9 CRC, 15 melanoma, 11 GIST) underwent simultaneous conventional mutation testing relevant to their tumour type (NSCLC pyrosequencing for *EGFR* L858R and *KRAS* codons 12/13/61 mutations and fragment analysis for *EGFR* exon 19 deletions, CRC and melanoma pyrosequencing for *BRAF* codon 600 mutations and *KRAS* codons 12/13/61 and GIST Sanger sequencing for *KIT* exon 11 deletions) and analysis on the Panel, the results of which are shown in S4 Tables 5A-D. The concordance between results with the Panel and conventional testing platforms is summarised in S3 Fig A-B.

With regards to the four standard diagnostic genes (*EGFR*, *KRAS*, *BRAF* and *KIT*), identical results were obtained for 77.8% (49/63) samples (includes samples with and without mutations); 34.9% (22/63) specimens had no mutations using either analysis, 1.6% (1/63) failed with both platforms and 41.3% (26/63) gave the same results with both methods, 76.9% (20/26) of which were single nucleotide variants (SNVs). Examination of the VAFs of the SNVs showed that both conventional testing and the Panel were able to detect mutations over a range of frequencies (conventional testing 5-90%, Panel 3.1-90.79%) indicating that the Panel is sufficiently sensitive to detect low level mutations. This is of particular relevance given that samples submitted for diagnostic testing are often contaminated with non-malignant tissue which will dilute any pathological mutations. The VAFs obtained using the two platforms were similar for the different SNVs with a mean difference of only 5.1% (SD 5.1). Across the different samples, the Panel gave slightly higher VAFs for mutations in *KRAS* and slightly lower VAFs for mutations in *BRAF* and *EGFR* compared to those obtained using pyrosequencing although concordance remains good across all the mutations examined in these genes (mean difference 3.9 SD 2.35 N=6, 6.44 SD 6.42 N=9, 5.83 SD 5.87 N=5 for *EGFR*, *KRAS* and *BRAF* respectively).

The initial concordance observed between detection of deletions in *EGFR* exon 19 and *KIT* exon 11 in NSCLC and GIST samples respectively was not as good as those observed in retrospective cohort 1 samples for SNVs (where sequencing with NGS was successful, 20/20 previously identified SNVs were correctly identified by the Panel): The default min-var-freq plugin parameters for indel calling originally employed in the bioinformatics pipeline only reported deletions with a frequency of 20% or higher meaning 2/4 *EGFR* exon 19 deletions (samples G121699T and G125878F) were missed due to low frequency. This problem was rectified by reducing the minimum reporting frequency to 1%. A further *EGFR* exon 19 deletion (sample G126936R) detected by fragment analysis was repeatedly not called by Ion Reporter yet was present when sequencing reads were subject to manual inspection in IGV. Investigation of this miscall revealed that the problem was related to the alignment algorithm and although not resolved during the analysis of retrospective cohort 1, modifications to this algorithm have subsequently been made such that Ion Reporter software from version 1.6.2 onwards is able to automatically detect the deletion.

Fifty seven percent (4/7) of deletions detected in *KIT* exon 11 amongst GIST specimens using Sanger sequencing were not reported using the Panel. In one sample this was due to complete failure in the analysis, but in the remaining samples visual inspection of the reads in IGV showed the discrepancy was due to poor coverage of the amplicon and the position of the deletion near the end of the amplicon causing problems with alignment. This systematic error which could not be resolved adequately bioinformatically has been eliminated in the updated commercially available version of the assay (Ion AmpliSeq Cancer Hotspot Panel v2) with a different design of the amplicon covering this region.

S3 Fig A-B demonstrate that although in 7.9% (5/63) of samples additional information was obtained using conventional genetic testing compared to the Panel (entirely accounted for by missed deletions in *EFGR* exon 19 and *KIT* exon 11 as described above), for 14.3% (9/63) of samples additional mutation information was obtained using the Panel compared to standard diagnostic testing: For 4/63 (6.3%) of samples conventional analysis failed whereas the Panel confirmed the absence of any mutations in the corresponding genes, while in the remaining 7.9% (5/63) of samples SNVs were detected using the Panel whereas they had been reported negative using conventional techniques. In 1/5 of these samples the point mutation (*BRAF* V600E in G127394L) was in a codon covered by conventional pyrosequencing while in the remainder they were outside the regions covered by diagnostic tests. In three of these samples at least one of the SNVs can be considered to be clinically actionable; G128225N NSCLC with *EGFR* G719A mutation – *EGFR* inhibition indicated^[[8]](#endnote-8)^, G127148B CRC with *KRAS* A146T mutation – should not receive anti-*EGFR* monoclonal antibody^[[9]](#endnote-9)^ and G127271X GIST with *KIT* N822K mutation – may be resistant to tyrosine kinase inhibition.^[[10]](#endnote-10)^

In total 32 SNVs were detected amongst 22 retrospective cohort 1 samples in genes or regions of genes which were not subject to routine diagnostic testing (9, 10, 12 and 1 mutations in 7, 7, 7 and 1 NSCLC, CRC, melanoma and GIST samples respectively). Thirty of these mutations underwent confirmatory testing using either pyro- or Sanger sequencing as shown in S3 Fig C and S5 Tables A-B. There was 100% concordance of those mutations tested using pyrosequencing with similar VAFs given using both methods (mean difference 4.15% SD 3.32 N=6). Of the 23 mutations which underwent confirmatory testing using Sanger sequencing 20 (87.0%) were confirmed: Those mutations not detected by this method all had VAFs on the Panel of <20% which can be considered to be the sensitivity of Sanger sequencing (G126332W *CSF1R* E317G VAF 6.67%, G126887K *TP53* R64X VAF 18.2% and G126731W *TP53* R81X VAF 8.1%).

**Comparison of concordance between mutations detected using the Cancer Panel and Sanger sequencing in retrospective cohort 2 samples**

Having used retrospective cohort 1 samples to make iterative adjustments to the bioinformatics pipeline as described above, the 45 samples from retrospective cohort 2 with previously identified mutations were analysed using the Panel. Forty samples originated from the VICTOR study and had previously been shown to contain SNVs within known hotspots of the *BRAF*, *FBXW7*, *KRAS*, *NRAS*, *PIK3CA* and *TP53* genes and five samples were historical control NSCLC and mesenchymal tumours known to contain indels in the *EGFR* and *KIT* genes respectively. Among retrospective cohort 2 samples, 98.3% (57/58) of previously identified mutations were confirmed using the Panel. A further 29 novel mutations were detected using the assay, 28 of which were tested using an alternative technique, with all being confirmed (S3 Fig D and S6 Tables A-C).

1. Midgley RS, McConkey CC, Johnstone EC, et al: [Phase III randomized trial assessing rofecoxib in the adjuvant setting of colorectal cancer: final results of the VICTOR trial.](http://www.ncbi.nlm.nih.gov/pubmed/20837956) J Clin Oncol 28:4575-4580, 2010 [↑](#endnote-ref-1)
2. Tie J, Lipton L, Desai J, et al: [KRAS mutation is associated with lung metastasis in patients with curatively resected colorectal cancer.](http://www.ncbi.nlm.nih.gov/pubmed/21239505) Clin Cancer Res 17:1122-1130, 2011 [↑](#endnote-ref-2)
3. Mouradov D, Domingo E, Gibbs P, et al: [Survival in stage II/III colorectal cancer is independently predicted by chromosomal and microsatellite instability, but not by specific driver mutations.](http://www.ncbi.nlm.nih.gov/pubmed/24042191) Am J Gastroenterol 108:1785-1793, 2013; [↑](#endnote-ref-3)
4. Robinson JT, Thorvaldsdottir H, Winckler W et al. Integrative genomics viewer. Nature Biotechnology 29:24-26, 2011 [↑](#endnote-ref-4)
5. Sherry ST, Ward M, Sirotkin K: [dbSNP-database for single nucleotide polymorphisms and other classes of minor genetic variation.](http://www.ncbi.nlm.nih.gov/pubmed/10447503) Genome Res 9:677-679, 1999 [↑](#endnote-ref-5)
6. 1000 Genomes: A Deep Catalog of Human Genetic Variation. <http://www.1000genomes.org/> [↑](#endnote-ref-6)
7. [Eckhart L](http://www.ncbi.nlm.nih.gov/pubmed?term=Eckhart%20L%5BAuthor%5D&cauthor=true&cauthor_uid=10814530), [Bach J](http://www.ncbi.nlm.nih.gov/pubmed?term=Bach%20J%5BAuthor%5D&cauthor=true&cauthor_uid=10814530), Ban J, et al: Melanin binds reversibly to thermostable DNA polymerase and inhibits its activity. [Biochem Biophys Res Commun](http://www.ncbi.nlm.nih.gov/pubmed/?term=L.+Eckhart%2C+J.+Bach%2C+J.+Ban%2C+E.+Tschachler+Melanin+binds+reversibly+to+thermostable+DNA+polymerase+and+inhibits+its+activity+Biochem.+Biophys.+Res.+Commun.) 271:726-730, 2000 [↑](#endnote-ref-7)
8. Han SW, Kim TY, Hwang PG, et al: [Predictive and prognostic impact of epidermal growth factor receptor mutation in non-small-cell lung cancer patients treated with gefitinib.](http://www.ncbi.nlm.nih.gov/pubmed/15710947) J Clin Oncol 23:2493-2501, 2005 [↑](#endnote-ref-8)
9. Stintzing S, Jung A, Rossius L, et al: Analysis of KRAS/NRAS and BRAF mutations in FIRE-3: A randomized phase III study of FOLFIRI plus cetuximab or bevacizumab as first-line treatment for wild-type (WT) KRAS (exon 2) metastatic colorectal cancer (mCRC) patients. Presented at the European Cancer Congress, Amsterdam, The Netherlands, September 27-October 1, 2013 [↑](#endnote-ref-9)
10. Heinrich MC, Corless CL, Blanke CD, et al: Molecular correlates of imatinib resistance in gastrointestinal stromal tumors. [J Clin Oncol](http://www.ncbi.nlm.nih.gov/pubmed/16954519)  24:4764-4774, 2006 [↑](#endnote-ref-10)
