# Supplementary material for: Clinical applicability and cost of a 46-gene panel for genomic analysis of solid tumours: Retrospective validation and prospective audit in the UK National Health Service
Source: PLoS Med. 2017 Feb 14;14(2):e1002230. doi: 10.1371/journal.pmed.1002230 (PMC5308858; doi:10.1371/journal.pmed.1002230)
Supplement: S2 Text — (DOCX) [file pmed.1002230.s018.docx]

COSTING QUESTIONNAIRE

**S2 Text: Costing Questionnaires**

Cancer Panel (NGS - 46 GENE PANEL)

CRC, MELANOMA and NSCLC

**GUIDANCE**

- This questionnaire is designed to collect resource use and cost data for next generation sequencing (NGS) with the Ion Torrent Personal Genome Machine (PGM) in the context of colorectal cancer, melanoma and non-small cell lung cancer.

- The questionnaire is divided into 6 stages, based on the DNA Quantification using the Qubit system Standard Operating Procedure (SOP) guide and the Ion Torrent PGM SOP guide. For each stage the tasks that must be performed are identified, based on the guides. If additional tasks are undertaken, please add extra rows to the tables, as required. If there are additional testing stages, please provide details of these stages in the blank ‘additional stage’ section.

- Please estimate all **staff** times in minutes. These estimates should reflect the amount of time that staff is actively engaged in a task i.e. not the total time for incubation, for example.

- All staff grades are assumed to be based on Agenda for Change pay scales. Please indicate if this is **NOT** the case.

- Information on **equipment** and **consumables** has been extracted from the guides and entered into the relevant tables for each stage. If additional equipment or consumables are required, please add extra rows to the tables, as required.

- Please enter all **equipment** and **consumable** costs and indicate if these costs include VAT. This questionnaire has been designed to collect the **costs** of consumables, equipment etc. to the laboratory, not the **prices** charged by the laboratory to those requesting tests.

- Please highlight any equipment which is **not** used solely for NGS, and estimate what percentage of that equipment’s time can be attributed to NGS.

- If the same piece of equipment is used across several stages (e.g. fridge), please include this at **every stage**, indicating that this is the case (e.g. by giving the piece of equipment the same name throughout i.e. fridge 1, fridge 2, etc.).

- For **consumables**, please specify both quantities and units e.g. 8 chips in a kit, 1 used per sample.

- Please indicate any stages which are **not necessary** for all test cases e.g. “x % of samples will have to be reprocessed as testing has failed due to human error”.

- Please also indicate when samples are **batched together** to undergo certain processes, along with the batch size.

1. How many samples are usually prepared at once?

*Describe how many samples are usually prepared at once*

# Stage 1. Isolation of genomic DNA from FFPE sections

Please skip stage 1 as it is included in the costing questionnaire for the Cobas platform.

# Stage 2. Sample reception

2. Please indicate the **staff** responsible for the tasks detailed below. If this can vary depending on availability/ case complexity, please indicate the grade of staff most likely to be responsible. Please also indicate the time staff is actively involved:

## Sample Reception

| Staff: *Choose an item*  Grade: *Choose an item* | |
| --- | --- |
| Task | Staff time (min) |
| Identify samples for cancer genetics and the source. |  |
| Check the sample and matching paperwork. |  |
| Identify the important information from the form for booking in. Stamp, allocate a lab 'G' number and re-transcribe the lab number on the sample. |  |

## HiCom Booking In

| Staff: *Choose an item*  Grade: *Choose an item* | |
| --- | --- |
| Task | Staff time (min) |
| Perform a HiCom search. If no previous records are found, create a new patient record. |  |
| Add a new sample and select the tests to be performed. Select the appropriate contact and save the record. |  |

## LIMS Booking In

| Staff: *Choose an item*  Grade: *Choose an item* | |
| --- | --- |
| Task | Staff time (min) |
| Log on LIMS and start requesting. Enter/check patient details. |  |
| Enter sample details using the allocated lab 'G' number, request the appropriate tests and book the sample in on LIMS. |  |

## Requesting Extraction and Filing

| Staff: *Choose an item*  Grade: *Choose an item* | |
| --- | --- |
| Task | Staff time (min) |
| Enter samples details on the extraction sheet and place the paperwork in the Cancer Genetics tray. |  |

##### Equipment

3. Please provide details of the pieces of **equipment** used to complete the tasks in **stage 2**. Some of these items have already been entered for you, based on the Ion Torrent SOP. If additional equipment is required, please add an additional row to the table and complete as appropriate:

| Type of equipment | Quantity required | Equipment cost (new) (£) | VAT included? | Annual maintenance cost (£)* | Predicted lifetime (years) |
| --- | --- | --- | --- | --- | --- |
| Computer |  |  | Yes No |  |  |
| Stamp |  |  | Yes No |  |  |
| *Other?* |  |  | Yes No |  |  |

** If applicable*

4. Are these pieces of equipment used entirely for targeted NGS?

Yes (Go to **Q7**)

No (Go to **Q5**)

5. If **No**, which pieces of equipment are used elsewhere in the laboratory? List pieces of equipment

6. What proportion (%) of their time or capacity is for targeted NGS? Enter percentage %

7. Are there any pieces of equipment that are used in **stage 2** which require samples to be batched together, rather than being processed one sample at a time?

Yes (Go to **Q8**)

No (Go to **Q10**)

8. If **Yes**, which pieces of equipment require samples to be batched together? List pieces of equipment

9. What would the standard batch size be? Enter batch size

##### Consumables

10. Please list the **consumables** used to complete the tasks in **stage 2**. If additional consumables are required, please add an additional row to the table and complete as appropriate:

| Type of consumable | Overall cost (e.g. £10 for a box of consumables X) | Number of units (e.g. 100 in one box) | Number of units used per sample (e.g. 2) |
| --- | --- | --- | --- |
|  |  |  |  |
|  |  |  |  |

11. What proportion (%) of samples will have to repeat **stage 2** due to error? *Enter percentage* %

# Stage 3. Ion Torrent - Library preparation

12. Please indicate the **staff** responsible for the tasks detailed below. If this can vary depending on availability/ case complexity, please indicate the grade of staff most likely to be responsible. Please also indicate the time staff is actively involved:

## Library Preparation

| Staff: *Choose an item*  Grade: *Choose an item* | |
| --- | --- |
| Task | Staff time (min) |
| Identify the samples that require Cancer Panel testing and fill in the Ion Torrent Library Prep record sheet. |  |
| Quantify the samples to be tested using the Qubit High Sensitivity Kit. |  |
| - Set up assay tubes and prepare the Qubit working solution and complete log sheet. |  |
| - Vortex all tubes for 2-3 seconds and incubate for 2 minutes. |  |
| - Turn on the Qubit system and insert the standard tubes. |  |
| - Take sample readings, save the data and record the concentration on the extraction worksheet. |  |
| When possible, dilute samples into a new and appropriately labelled tube. |  |
| Ensure that the reagents are thoroughly thawed and defrosted. Prepare the PCR reaction mix. |  |
| Label PCR tubes and dispense reaction mix into the tubes and add the correct volume of sample to the appropriate tube. |  |
| Place the tubes into a thermo cycler, and start the cycling program. Following completion of the PCR, store samples. |  |

## Barcoding and Library Purification

| Staff: *Choose an item*  Grade: *Choose an item* | |
| --- | --- |
| Task | Staff time (min) |
| Add FuPa reagent to each amplified sample. Load the tubes in the thermal cycler and start the digestion program. |  |
| For each sample select a different barcode and record on the log sheet. Retrieve the barcodes and adapter to be used and leave them to thaw on ice. |  |
| For each barcode X chosen, prepare a mix of Ion P1 Adapter and Ion Xpress Barcode X for each adapter. Add Switch Solution, DNA Ligase and the appropriate diluted barcode. |  |
| Load the tubes in the thermal cycler and start the ligation program. |  |
| Label eppendorfs and add Axyprep beads to each tube. For each sample, transfer the library preparation in the corresponding tube. Incubate the mixture. |  |
| Place the eppendorfs in a magnetic rack and incubate. |  |
| Add 70% ethanol and move the tubes from side to side on the magnetic racks to wash the beads, then remove and discard the supernatant. Repeat ethanol wash. |  |
| Keep the eppendorfs on the magnetic rack, air-dry the beads until all ethanol has evaporated. Remove the tubes from the magnetic rack and add Low TE to the pellet. |  |
| Place eppendorfs back in magnetic rack. Remove supernatant from eppendorf, transfer to new eppendorf and store samples. |  |

## Library Quantification

| Staff: *Choose an item*  Grade: *Choose an item* | |
| --- | --- |
| Task | Staff time (min) |
| Prepare three 10‐fold serial dilutions of the E. coli DH10B Ion Control Library. |  |
| Prepare reaction mixtures. Dispense aliquots into the wells of a PCR plate. Add diluted library samples to each well in duplicate. |  |
| Program the ABI real‐time instrument. Enter the concentrations of the control library standards. |  |
| Following qPCR, calculate the average concentration of the undiluted libraries and report any unamplified library. Prepare dilutions of libraries. |  |

##### Equipment

13. Please provide details of the pieces of **equipment** used to complete the tasks in **stage 3**. Some of these items have already been entered for you, based on the Ion Torrent SOP. If additional equipment is required, please add an additional row to the table and complete as appropriate:

| Type of equipment | Quantity required | Equipment cost (new) (£) | VAT included? | Annual maintenance cost (£)* | Predicted lifetime (years) |
| --- | --- | --- | --- | --- | --- |
| ABI real‐time instrument |  |  | Yes No |  |  |
| Magnetic rack |  |  | Yes No |  |  |
| PCR plate |  |  | Yes No |  |  |
| Thermal cycler |  |  | Yes No |  |  |
| UV Hood |  |  | Yes No |  |  |
| *Other?* |  |  | Yes No |  |  |

** If applicable*

14. Are these pieces of equipment used entirely for targeted NGS?

Yes (Go to **Q17**)

No (Go to **Q15**)

15. If **No**, which pieces of equipment are used elsewhere in the laboratory? List pieces of equipment

16. What proportion (%) of their time or capacity is for targeted NGS? Enter percentage %

17. Are there any pieces of equipment that are used in **stage 3** which require samples to be batched together, rather than being processed one sample at a time?

Yes (Go to **Q18**)

No (Go to **Q20**)

18. If **Yes**, which pieces of equipment require samples to be batched together? List pieces of equipment

19. What would the standard batch size be? Enter batch size

##### Consumables

20. Please list the **consumables** used to complete the tasks in **stage 3**. Some of these consumables have already been entered for you, based on the Ion Torrent SOP. If additional consumables are required, please add an additional row to the table and complete as appropriate:

| Type of consumable | Overall cost (e.g. £10 for a box of consumables X) | Number of units (e.g. 100 in one box) | Number of units used per sample (e.g. 2) |
| --- | --- | --- | --- |
| 70% ethanol |  |  |  |
| Axyprep beads |  |  |  |
| Eppendorfs |  |  |  |
| Ion AmpliSeq Library Kit 2.0 (Part NO 4475345) | £511.2 |  |  |
| Ion P1 Adapter and Ion Xpress Barcode X Kit |  |  |  |
| - 2X TaqMan MasterMix |  |  |  |
| - 20X Ion TaqMan Assay |  |  |  |
| - DNA Ligase |  |  |  |
| - FAM dye/MGB (TaqMan probe reporter) |  |  |  |
| - FuPa |  |  |  |
| - PCR reaction mix |  |  |  |
| - ROX Reference Dye |  |  |  |
| - Serial dilutions of the E. Coli DH10B Ion Control Library |  |  |  |
| - Switch Solution |  |  |  |
| PCR tubes |  |  |  |
| qPCR Ion Library Quantitation Kit (Part NO 4468802) | £752 |  |  |
| Qubit High Sensitivity Kit |  |  |  |
| - Qubit Assay Tubes |  |  |  |
| - Qubit working solution |  |  |  |
| *Other?* |  |  |  |

21. What proportion (%) of samples will have to repeat **stage 3** due to error? *Enter percentage* %

# Stage 4. Ion OneTouch and OneTouch ES protocol

22. Please indicate the **staff** responsible for the tasks detailed below. If this can vary depending on availability/ case complexity, please indicate the grade of staff most likely to be responsible. Please also indicate the time staff is actively involved:

## Ion OneTouch Configuration

| Staff: *Choose an item*  Grade: *Choose an item* | |
| --- | --- |
| Task | Staff time (min) |
| Complete a record sheet for the OneTouch and PGM assay. Remove the OneTouch Plus Reaction Filter Assembly and install the OneTouch 200 Plus Amplification Plate. |  |
| Fill the OneTouch Reagent Tube with OneTouch Oil. Remove the tube and agitate the tube. Invert the OneTouch Oil bottle and fill the Reagent tube with Oil. Insert the filled Reagent Tube back. |  |
| Fill the OneTouch Reagent Tube with OneTouch Recovery Solution. Invert the bottle of Recovery Solution and fill the Reagent tube quarter full. Insert the Reagent Tube back into place. If solution is not clear, gently warm in a container containing warm tap water. |  |
| Insert a Recovery Tube in the centrifuge. Remove and empty waste. Then place the container back. |  |

## Prepare and Install Amplification Solution

| Staff: *Choose an item*  Grade: *Choose an item* | |
| --- | --- |
| Task | Staff time (min) |
| Dilute each library. Multiplex the libraries into an eppendorf and add the diluted libraries. |  |
| Prepare the reagents and add the components in an eppendorf. |  |
| Vortex the ISPs, pipet the ISPs and add the ISPs to the amplification solution. |  |
| Obtain an OneTouch Plus Reaction Filter Assembly and place it into a tube rack. Add the amplification solution through the sample port. First, vortex the amplification solution and then centrifuge. Pipet the amplification solution and insert the tip into the sample port. |  |
| Add OneTouch Reaction Oil. Pipet the Reaction Oil. If necessary, dab a disposable wiper around the ports to remove any liquid. |  |
| Invert the OneTouch Plus Reaction Filter Assembly. Lift out the Filter Assembly from the tube rack and insert the 3 ports of the Filter Assembly into the three holes of the OneTouch Instrument. Start the assisted Run. |  |

## Recovering the template-positive ISP

| Staff: *Choose an item*  Grade: *Choose an item* | |
| --- | --- |
| Task | Staff time (min) |
| Ensure that the samples are centrifuged. If not, centrifuge again. |  |
| Remove and discard the Recovery Router. Remove both Recovery Tubes from the instrument and label a tube for the template-positive ISPs. |  |
| Pipette Recovery Solution from Recovery Tube. Resuspend the template-positive ISPs in the remaining Recovery Solution and transfer each suspension to the new labelled tube. |  |
| Bring the combined suspensions in the new labelled tube and centrifuge the template-positive ISP suspension. |  |
| Remove supernatant and vortex the pellet. |  |
| Enrich the template-positive ISPs |  |

## Maintaining the OneTouch Instrument

| Staff: *Choose an item*  Grade: *Choose an item* | |
| --- | --- |
| Task | Staff time (min) |
| Check the level of OneTouch Oil and OneTouch Recovery Solution. If needed, add Oil and/or Recovery Solution. |  |
| Ensure that the used Ion OneTouch™ Plus Reaction Filter Assembly is still in place, and install an empty cap below the sample injector. |  |
| Remove and dispose the used amplification plate from the instrument. |  |
| Insert the Adaptor. |  |
| Clean Ion OneTouch. At the end of the cleaning run, remove cleaning adapter and disconnect it. Remove the cap from the centrifuge. Wipe the residue from the centrifuge lid and centre sample injector tubing. Inspect the sample injector tube after cleaning and close the lid. |  |

## Enrichment of Template-positive ISPs

| Staff: *Choose an item*  Grade: *Choose an item* | |
| --- | --- |
| Task | Staff time (min) |
| Prepare fresh Melt-Off Solution. |  |
| Add MyOne Beads Wash Solution to an eppendorf. Vortex the bottle containing the Dynabeads MyOne Streptavidin C1 Beads. Place the tube on a magnet, remove and discard the supernatant. |  |
| Add MyOneBeads Wash Solution and vortex and centrifuge the tube. Centrifuge the ISPs, remove the supernatant and add sufficient Ion OneTouch Wash Solution. |  |
| Pipet the ISPs, then transfer resuspended ISPs in Ion OneTouch Wash Solution into the Wells of the 8-well strip. |  |
| Load a new tip in the Tip Arm and prepare the Ion OneTouch ES. Insert a new, opened PCR tube into the hole in the base of the Tip Loader and load the 8-well strip. |  |
| Pipet the contents of Well 2. Turn on the Ion OneTouch ES and start the run. |  |
| After the run, close and remove the PCR tube containing the enriched ISPs. Prepare the Ion OneTouch ES. First, load a new tip in the Tip Arm. |  |

## Remove and Wash the ISPs

| Staff: *Choose an item*  Grade: *Choose an item* | |
| --- | --- |
| Task | Staff time (min) |
| Centrifuge the PCR tube containing the enriched ISPs. |  |
| Remove supernatant and add Ion OneTouch Wash Solution. Pipet the solution. Centrifuge the PCR tube. |  |
| Remove supernatant. Add Ion OneTouch Wash and pipet. If beads are present; pipet, place the PCR tube against a magnet, transfer the supernatant to a new PCR tube, centrifuge the supernatant, and then remove supernatant. |  |
| Remove the used tip, discard the used 8-well strip and store material. |  |

##### Equipment

23. Please provide details of the pieces of **equipment** used to complete the tasks in **stage 4**. Some of these items have already been entered for you, based on the Ion Torrent SOP. If additional equipment is required, please add an additional row to the table and complete as appropriate:

| Type of equipment | Quantity required | Equipment cost (new) (£) | VAT included? | Annual maintenance cost (£)* | Predicted lifetime (years) |
| --- | --- | --- | --- | --- | --- |
| 250-mL bottle |  |  | Yes No |  |  |
| Adapter |  |  | Yes No |  |  |
| Centrifuge |  |  | Yes No |  |  |
| DynaMag-2 magnet |  |  | Yes No |  |  |
| Fridge |  |  | Yes No |  |  |
| One Touch |  |  | Yes No |  |  |
| One Touch ES |  |  | Yes No |  |  |
| Pipet |  |  | Yes No |  |  |
| Tube rack |  |  | Yes No |  |  |
| Vortexer |  |  | Yes No |  |  |
| *Other?* |  |  | Yes No |  |  |

** If applicable*

24. Are these pieces of equipment used entirely for targeted NGS?

Yes (Go to **Q27**)

No (Go to **Q25**)

25. If **No**, which pieces of equipment are used elsewhere in the laboratory? List pieces of equipment

26. What proportion (%) of their time or capacity is for targeted NGS? Enter percentage %

27. Are there any pieces of equipment that are used in **stage 4** which require samples to be batched together, rather than being processed one sample at a time?

Yes (Go to **Q28**)

No (Go to **Q30**)

28. If **Yes**, which pieces of equipment require samples to be batched together? List pieces of equipment

29. What would the standard batch size be? Enter batch size

##### Consumables

30. Please list the **consumables** used to complete the tasks in **stage 4**. Some of these consumables have already been entered for you, based on the Ion Torrent SOP. If additional consumables are required, please add an additional row to the table and complete as appropriate:

| Type of consumable | Overall cost (e.g. £10 for a box of consumables X) | Number of units (e.g. 100 in one box) | Number of units used per sample (e.g. 2) |
| --- | --- | --- | --- |
| Disposable wipers |  |  |  |
| Dynabeads MyOne Streptavidin C1 Beads |  |  |  |
| Eppendorf |  |  |  |
| Ion PGM Template OneTouch 200 Kit v2 (Part NO 4480974) | £797.6 |  |  |
| - 8-well strip |  |  |  |
| - Amplification Plate |  |  |  |
| - MyOne Beads Wash Solution |  |  |  |
| - OneTouch Oil |  |  |  |
| - OneTouch Plus Reaction Filter Assembly |  |  |  |
| - OneTouch Recovery Solution |  |  |  |
| - Reagent Tube |  |  |  |
| - Recovery Router |  |  |  |
| - Tip |  |  |  |
| Melt-Off Solution (mix of NaOH and Tween 20) |  |  |  |
| PCR tube |  |  |  |
| Recovery Tubes |  |  |  |
| *Other?* |  |  |  |

31. What proportion (%) of samples will have to repeat **stage 4** due to error? *Enter percentage* %

# Stage 5. Ion Torrent - Sequencing

32. Please indicate the **staff** responsible for the tasks detailed below. If this can vary depending on availability/ case complexity, please indicate the grade of staff most likely to be responsible. Please also indicate the time staff is actively involved:

## Creating Run Plan

| Staff: *Choose an item*  Grade: *Choose an item* | |
| --- | --- |
| Task | Staff time (min) |
| Set up a run plan for each chip by accessing the Ion Torrent server and add a plan. Fill out the information and save the plan. |  |

## PGM – Maintenance

| Staff: *Choose an item*  Grade: *Choose an item* | |
| --- | --- |
| Task | Staff time (min) |
| Attach cleaning bottles on the instrument, empty any remaining solution from each cleaning bottle and rinse each bottle twice, and add water to a cleaning bottle. Rinse the outside of the Sipper Tube in the W1 position with a squirt bottle, and attach the bottle to the W1 position on the Ion PGM System. |  |
| Follow the instructions on the touchscreen to perform the cleaning procedure. When prompted, remove the W1 cleaning bottle, then rinse and reinstall the cleaning bottle filled with water. After cleaning, proceed to initialization. |  |
| Fill a glass bottle with water and add a PGM Cleaning Tablet. When the tablet has dissolved, add NaOH and filter the solution. Select Clean PGM on the touchscreen. |  |
| Add filtered chlorite solution to a cleaning bottle. Rinse the outside of the Sipper Tube, and attach the bottle to the W1 position on the Ion PGM System. |  |
| Follow the instructions on the touchscreen to perform the cleaning procedure. When prompted, remove the W1 cleaning bottle, rinse the outside of the sipper, then install a cleaning bottle. |  |

## PGM – Initialization

| Staff: *Choose an item*  Grade: *Choose an item* | |
| --- | --- |
| Task | Staff time for activity (min) |
| Prepare the Wash and Reagent Bottles. Warm the 10X W3 Solution and shake the bottle. |  |
| Remove the dNTP stock solutions from the freezer and thaw on ice. |  |
| Check the tank pressure. If needed, change tank. |  |
| Prepare the Wash 2 Bottle, and place a magnetic stir bar into the empty bottle. |  |
| Insert the argon gas tube into the empty Wash 2 Bottle, set the flow meter, and flow gas into the bottle, cap the bottle and add water. |  |
| Place the Wash 2 Bottle on a stir plate and set the gas flow. Add PGM 200 W2 Solution to the Wash 2 Bottle while the gas is flowing. When completed remove the gas tube and cap the bottle. |  |
| Prepare, rinse, wash the Wash 1 and Wash 3 Bottles, and install bottles and Sippers. |  |
| Verify the gas pressure. If low, retry gas-pressure verification. |  |
| Prepare Reagent Bottles. After each dNTP stock solution has thawed, vortex to mix and centrifuge to collect the contents. |  |
| Label four new Reagent Bottles. Transfer each dNTP stock solution into the Reagent Bottles. Attach the Sipper Tubes and Reagent Bottles. |  |
| After the wash solutions have initialized, follow the on-screen prompts to remove the used Sipper Tubes and collection trays from the dNTP ports. Change gloves. |  |
| Insert a new Sipper Tube into each dNTP port. Attach each Reagent Bottle to the Ion PGM System. Complete initialization. |  |

## Sample Preparation

| Staff: *Choose an item*  Grade: *Choose an item* | |
| --- | --- |
| Task | Staff time (min) |
| Add Control Ion Sphere Particles and anneal Sequencing Primer to the enriched ISPs. Vortex the Control Ion Sphere Particles and centrifuge. |  |
| Add Control Ion Sphere Particles to the enriched, template-positive ISPs in a PCR tube. Add Annealing Buffer. Centrifuge the tube. |  |
| Remove the supernatant and add Sequencing Primer. Add Annealing Buffer if necessary. Pipet the sample. |  |
| Program a thermal cycler for 95°C for 2 minutes and then 37°C for 2 minutes. Place the tube and run the program. Once the cycling finishes, add PGM 200 Sequencing polymerase and incubate. |  |

## Chip Preparation

| Staff: *Choose an item*  Grade: *Choose an item* | |
| --- | --- |
| Task | Staff time (min) |
| Label a new chip and place it on the Ion PGM System grounding plate or in the Ion centrifuge adapter/rotor bucket. |  |
| Prepare the Ion PGM System to test a new Ion Chip. Scan the barcode. Perform a Chip Check. Check for leaks on the chip. |  |
| When Chip Check is complete, remove the chip. If failed, re-seat the chip and calibrate to repeat. Replace the chip from the socket with a used chip. Place the Ion Chip in the centrifuge adapter bucket. |  |
| Using a Rainin SR-L200F pipette tip, slowly and steadily pipet 100% isopropanol into the large loading port of the Chip, then remove and discard any displaced liquid from the other port. |  |
| Add Annealing Buffer into the large loading port of the Chip, then remove and discard any displaced liquid from the other port. Repeat one more time. |  |

## Sample Loading

| Staff: *Choose an item*  Grade: *Choose an item* | |
| --- | --- |
| Task | Staff time (min) |
| Tilt the chip 45 degrees and insert the pipette tip into the loading port. Remove Annealing Buffer. |  |
| Place the washed chip in the centrifuge adapter bucket, and transfer the bucket to the MiniFuge. Centrifuge. Transfer the chip to the grounding plate on the Ion PGM Sequencer and wipe off any liquid remaining on the centrifuge bucket. Transfer the chip to the centrifuge adapter bucket. |  |
| Following polymerase incubation, collect the entire sample into a Rainin SR-L200F pipette tip and insert the tip into the loading port of the chip. Deposit the ISPs. Remove and discard any displaced liquid from the other port of the chip. |  |
| Transfer the chip to the MiniFuge. Centrifuge and then remove the chip from the centrifuge bucket. |  |
| Mix the sample in the chip. Tilt the chip 45 degrees and insert the pipette tip into the loading port. Pipet the sample in and out of the chip. Centrifuge the chip. Repeat the chip mixing three more time, then spin. |  |
| Tilt the chip at a 45-degree angle and remove liquid from the loading port. Discard the liquid. If liquid remains in the chip, perform a quick spin. Load and clamp the chip on the PGM and empty the waste container. |  |

## Sequencing Run

| Staff: *Choose an item*  Grade: *Choose an item* | |
| --- | --- |
| Task | Staff time (min) |
| Select Run Plan and confirm or adjust settings. |  |
| Check the chip for leaks before closing the lid. Calibrate the chip. If passed, perform the run and monitor. |  |

##### Equipment

33. Please provide details of the pieces of **equipment** used to complete the tasks in **stage 5**. Some of these items have already been entered for you, based on the Ion Torrent SOP. If additional equipment is required, please add an additional row to the table and complete as appropriate:

| Type of equipment | Quantity required | Equipment cost (new) (£) | VAT included? | Annual maintenance cost (£)* | Predicted lifetime (years) |
| --- | --- | --- | --- | --- | --- |
| Adapter bucket and Minifuge |  |  | Yes No |  |  |
| Centrifuge |  |  | Yes No |  |  |
| Computer |  |  | Yes No |  |  |
| Freezer |  |  | Yes No |  |  |
| Glass bottle |  |  | Yes No |  |  |
| Ice machine |  |  | Yes No |  |  |
| Ion PGM Sequencer |  |  | Yes No |  |  |
| - Argon Gas tube |  |  | Yes No |  |  |
| - Barcode scanner |  |  | Yes No |  |  |
| - Ion Torrent server |  |  | Yes No |  |  |
| - Wash bottles |  |  | Yes No |  |  |
| Magnetic stir bar |  |  | Yes No |  |  |
| Squirt bottle |  |  | Yes No |  |  |
| Stir plate |  |  | Yes No |  |  |
| Tank, gas regulator |  |  | Yes No |  |  |
| Thermal cycler |  |  | Yes No |  |  |
| Vortexer |  |  | Yes No |  |  |
| *Other?* |  |  | Yes No |  |  |

** If applicable*

34. Are these pieces of equipment used entirely for targeted NGS?

Yes (Go to **Q37**)

No (Go to **Q35**)

35. If **No**, which pieces of equipment are used elsewhere in the laboratory? List pieces of equipment

36. What proportion (%) of their time or capacity is for targeted NGS? Enter percentage %

37. Are there any pieces of equipment that are used in **stage 5** which require samples to be batched together, rather than being processed one sample at a time?

Yes (Go to **Q38**)

No (Go to **Q40**)

38. If **Yes**, which pieces of equipment require samples to be batched together? List pieces of equipment

39. What would the standard batch size be? Enter batch size

##### Consumables

40. Please list the **consumables** used to complete the tasks in **stage 5**. Some of these consumables have already been entered for you, based on the Ion Torrent SOP. If additional consumables are required, please add an additional row to the table and complete as appropriate:

| Type of consumable | Overall cost (e.g. £10 for a box of consumables X) | Number of units (e.g. 100 in one box) | Number of units used per sample (e.g. 2) |
| --- | --- | --- | --- |
| 100% isopropanol |  |  |  |
| Chip Kit (Part NO 4469496) | £1510.4 |  |  |
| Gloves |  |  |  |
| Ion AmpliSeq Primer Pool (Part NO 4471262) | £160 |  |  |
| Ion PGM 200 Sequencing Kit (Part NO 4474004) | £640.8 |  |  |
| - 10X W3 Solution |  |  |  |
| - Annealing Buffer |  |  |  |
| - dNTP stock solutions |  |  |  |
| - PGM Cleaning Tablet (chlorite solution) |  |  |  |
| - Cleaning bottles |  |  |  |
| - Conical tube |  |  |  |
| - Control Ion Sphere Particles |  |  |  |
| - Labels |  |  |  |
| - PGM 200 W2 Solution |  |  |  |
| - Reagent bottles |  |  |  |
| - Sipper Tube |  |  |  |
| NaOH solution |  |  |  |
| PCR tube |  |  |  |
| Rainin SR-L200F pipette tip |  |  |  |
| *Other?* |  |  |  |

41. What proportion (%) of samples will have to repeat **stage 5** due to error? *Enter percentage* %

# STAGE 6. Interpretation and Reporting

42. Please indicate the **staff** responsible for the tasks detailed below. If this can vary depending on availability/ case complexity, please indicate the grade of staff most likely to be responsible. Please also indicate the time staff is actively involved:

| Task | Staff type | Staff grade | Staff time (min) |
| --- | --- | --- | --- |
| Results are uploaded to the Ion Torrent server. | *Choose an item* | *Choose an item* |  |
| Results are analysed for ‘noise’ by Ion Reporter Software. | *Choose an item* | *Choose an item* |  |
| Results are collated and an initial report is prepared from the HiCom laboratory system. | *Choose an item* | *Choose an item* |  |
| Uncommon mutations are explored in the catalogue of somatic mutations in cancer (COSMIC). | *Choose an item* | *Choose an item* |  |
| The report is reviewed by a senior clinical scientist. | *Choose an item* | *Choose an item* |  |
| Results are entered on the haematology internal laboratory reporting system (LIMS) for invoicing. | *Choose an item* | *Choose an item* |  |
| Results are reported and counter-signed by senior clinical scientist before being sent away. | *Choose an item* | *Choose an item* |  |

##### Equipment

43. Please provide details of the pieces of **equipment** used to complete the tasks in **stage 6**. Some of these items have already been entered for you, based on the Ion Torrent SOP. If additional equipment is required, please add an additional row to the table and complete as appropriate:

| Type of equipment | Quantity required | Equipment cost (new) (£) | VAT included? | Annual maintenance cost (£)* | Predicted lifetime (years) |
| --- | --- | --- | --- | --- | --- |
| Ion Reporter Software |  |  | Yes No |  |  |
| *Other?* |  |  | Yes No |  |  |

** If applicable*

44. Are these pieces of equipment used entirely for targeted NGS?

Yes (Go to **Q47**)

No (Go to **Q45**)

45. If **No**, which pieces of equipment are used elsewhere in the laboratory? List pieces of equipment

46. What proportion (%) of their time or capacity is for targeted NGS? Enter percentage %

47. Are there any pieces of equipment that are used in **stage 6** which require samples to be batched together, rather than being processed one sample at a time?

Yes (Go to **Q48**)

No (Go to **Q50**)

48. If **Yes**, which pieces of equipment require samples to be batched together? List pieces of equipment

49. What would the standard batch size be? Enter batch size

##### Consumables

50. Please list the **consumables** used to complete the tasks in **stage 6**. If needed, add additional rows to the table and complete as appropriate:

| Name | Overall cost (e.g. £10 for a box of consumables X) | Number of units (e.g. 100 in one box) | Number of units used per sample (e.g. 2) |
| --- | --- | --- | --- |
|  |  |  |  |
|  |  |  |  |

51. What proportion (%) of samples will have to repeat **stage 6** due to error? *Enter percentage* %

# ADDITIONAL STAGE (IF REQUIRED)

52. Please indicate the **staff** responsible for the tasks detailed below. If this can vary depending on availability/ case complexity, please indicate the grade of staff most likely to be responsible. Please also indicate the time staff is actively involved:

| Task | Staff type | Staff grade | Staff time (min) |
| --- | --- | --- | --- |
|  |  |  |  |
|  |  |  |  |

##### Equipment

53. Please provide details of the pieces of **equipment** used to complete the tasks in **this additional stage**. If additional equipment is required, please add an additional row to the table and complete as appropriate:

| Type of equipment | Quantity required | Equipment cost (new) (£) | VAT included? | Annual maintenance cost (£)* | Predicted lifetime (years) |
| --- | --- | --- | --- | --- | --- |
|  |  |  | Yes No |  |  |
|  |  |  | Yes No |  |  |

** If applicable*

54. Are these pieces of equipment used entirely for targeted NGS?

Yes (Go to **Q57**)

No (Go to **Q55**)

55. If **No**, which pieces of equipment are used elsewhere in the laboratory? List pieces of equipment

56. What proportion (%) of their time or capacity is for targeted NGS? Enter percentage %

57. Are there any pieces of equipment that are used in **this additional stage** which require samples to be batched together, rather than being processed one sample at a time?

Yes (Go to **Q58**)

No (Go to **Q60**)

58. If **Yes**, which pieces of equipment require samples to be batched together? List pieces of equipment

59. What would the standard batch size be? Enter batch size

##### Consumables

60. Please list the **consumables** used to complete the tasks in **this additional stage**:

| Type of consumable | Overall cost (e.g. £10 for a box of consumables X) | Number of units (e.g. 100 in one box) | Number of units used per sample (e.g. 2) |
| --- | --- | --- | --- |
|  |  |  |  |
|  |  |  |  |

61. What proportion (%) of samples will have to repeat **this additional stage** due to error? *Enter percentage* %

# MISCELLANEOUS

62. Are there any other costs that are incurred in undertaking targeted NGS which are not specific to any of the above stages e.g. IT equipment and stationery? If needed, add additional rows to the table and complete as appropriate:

| Type of equipment | Quantity required | Equipment cost (new) (£) | VAT included? | Annual maintenance cost (£)* | Predicted lifetime (years) |
| --- | --- | --- | --- | --- | --- |
| Maintenance contract |  |  | Yes No |  |  |
| Software license Life Technologies GeneAnalyzer |  |  | Yes No |  |  |
| Training and on-going support for using GeneAnalyzer |  |  | Yes No |  |  |
| *Other?* |  |  | Yes No |  |  |

** If applicable*

COSTING QUESTIONNAIRE

COBAS PLATFORM (REAL-TIME PCR: BRAF, KRAS and EGFR)

CRC, MELANOMA and NSCLC

**GUIDANCE**

- This questionnaire is designed to collect resource use and cost data for real-time PCR with the Cobas 4800 in the context of colorectal cancer, melanoma and non-small cell lung cancer.

- The questionnaire is divided into 6 stages, based on the DNA Extraction Protocol and the Cobas 4800 Standard Operation Procedure (SOP) guide. For each stage the tasks that must be performed are identified, based on the protocol and guide. If additional tasks are undertaken, please add extra rows to the tables, as required. If there are additional testing stages, please provide details of these stages in the blank ‘additional stage’ section.

- Please estimate all **staff** times in minutes. These estimates should reflect the amount of time that staff is actively engaged in a task i.e. not the total time for incubation, for example.

- All staff grades are assumed to be based on Agenda for Change pay scales. Please indicate if this is **NOT** the case.

- Information on **equipment** and **consumables** has been extracted from the protocol and guides and entered into the relevant tables for each stage. If additional equipment or consumables are required, please add extra rows to the tables, as required.

- Please enter all **equipment** and **consumable** costs and indicate if these costs include VAT. This questionnaire has been designed to collect the **costs** of consumables, equipment etc. to the laboratory, not the **prices** charged by the laboratory to those requesting tests.

- Please highlight any equipment which is **not** used solely for real-time PCR, and estimate what percentage of that equipment’s time can be attributed to real-time PCR.

- If the same piece of equipment is used across several stages (e.g. fridge), please include this at **every stage**, indicating that this is the case (e.g. by giving the piece of equipment the same name throughout i.e. fridge 1, fridge 2, etc.).

- For **consumables**, please specify both quantities and units e.g. 12 slides in a box, 1 used per sample.

- Please indicate any stages which are **not necessary** for all test cases e.g. “x % of samples will have to be reprocessed as testing has failed due to human error”.

- Please also indicate when samples are **batched together** to undergo certain processes, along with the batch size.

1. How many samples are usually prepared at once?

*Describe how many samples are usually prepared at once*

# Stage 1. Isolation of genomic DNA from FFPE sections

2. Please indicate the **staff** responsible for the tasks detailed below. If this can vary depending on availability/ case complexity, please indicate the grade of staff most likely to be responsible. Please also indicate the time staff is actively involved:

| Staff: *Choose an item*  Grade: *Choose an item* | |
| --- | --- |
| Task | Staff time (min) |
| Remove paraffin, prepare the extraction record worksheet, label eppendorf tubes. Scrape off the tissue from slides and place into the labelled eppendorf tubes. |  |
| Pulse spin samples, a**dd Qiagen Deparaffinization Solution to each sample and vortex. Then pulse spin samples again.** |  |
| **Incubate and then cool.** |  |
| **Add Buffer ATL to each sample vortex and centrifuge. Add proteinase K, mix and secure the eppendorf lid with parafilm.** |  |
| **Incubate while preparing three sets of labelled elution eppendorf tubes.** |  |
| **Centrifuge tubes after incubation and transfer the lower, clear phase into an eppendorf tube. Record this on the extraction worksheet.** |  |
| **Pre-mix equal quantities of Buffer AL and ethanol (96-100%) and vortex. Add AL/EtOH mix to sample, vortex/pipette and quick spin.** |  |
| **Transfer lysate to labelled QIAamp MinElute column and centrifuge. Add Buffer AW1, centrifuge and transfer column to a collection tube. Add Buffer AW2, centrifuge and transfer column to a collection tube. Empty the flow-through and re-centrifuge if necessary. Centrifuge.** |  |
| **Line up MinElute columns and get a second person to confirm the correct labelling for each sample. Record this witness step on the extraction worksheet. Place each column into its corresponding elution tube and discard the collection tube containing the flow-through.** |  |
| **Apply Buffer ATE to the centre of the membrane and incubate. Then centrifuge.** |  |
| **Transfer each column, repeat the line-up of all MinElute columns and discard the collection tube containing the flow-through.** |  |
| **Quantify the DNA concentrations. Save the data on a memory stick and write the DNA concentrations on the extraction worksheet. Store FFPE DNA until use.** |  |

##### Equipment

3. Please provide details of the pieces of **equipment** used to complete the tasks in **stage 1**. Some of these items have already been entered for you, based on the DNA extraction protocol for the Cancer Genetics Service. If additional equipment is required, please add an additional row to the table and complete as appropriate:

| Type of equipment | Quantity required | Equipment cost (new) (£) | VAT included? | Annual maintenance cost (£)* | Predicted lifetime (years) |
| --- | --- | --- | --- | --- | --- |
| Freezer |  |  | Yes No |  |  |
| Fridge |  |  | Yes No |  |  |
| Heating block |  |  | Yes No |  |  |
| Laboratory coat |  |  | Yes No |  |  |
| Micro centrifuge |  |  | Yes No |  |  |
| Pipettes |  |  | Yes No |  |  |
| Qubit 2.0 fluorometer |  |  | Yes No |  |  |
| Scalpels |  |  | Yes No |  |  |
| Vortexer |  |  | Yes No |  |  |
| *Other?* |  |  | Yes No |  |  |

** If applicable*

4. Are these pieces of equipment used entirely for real-time PCR?

Yes (Go to **Q7**)

No (Go to **Q5**)

5. If **No**, which pieces of equipment are used elsewhere in the laboratory? *List pieces of equipment*

6. What proportion (%) of their time or capacity is for real-time PCR? *Enter percentage* %

7. Are there any pieces of equipment that are used in **stage 1** which require samples to be batched together, rather than being processed one sample at a time?

Yes (Go to **Q8**)

No (Go to **Q10**)

8. If **Yes**, which pieces of equipment require samples to be batched together? *List pieces of equipment*

9. What would the standard batch size be? *Enter batch size*

##### Consumables

10. Please list the **consumables** used to complete the tasks in **stage 1**. Some of these consumables have already been entered for you, based on the DNA extraction protocol for the Cancer Genetics Service. If additional consumables are required, please add an additional row to the table and complete as appropriate:

| Type of consumable | Overall cost (e.g. £10 for a box of consumables X) | Number of units (e.g. 100 in one box) | Number of units used per sample (e.g. 2) |
| --- | --- | --- | --- |
| Aerosol-barrier filter pipette tips |  |  |  |
| dsDNA HS Assay Kit |  |  |  |
| Eppendorf tubes |  |  |  |
| Ethanol (96-100%) (BDH Product code 10107) |  |  |  |
| Micro centrifuge tubes |  |  |  |
| Protective gloves |  |  |  |
| QIAamp DNA FFPE Tissue Kit (Product code 56404) |  |  |  |
| - Buffer AW1 concentrate |  |  |  |
| - Buffer AW2 concentrate |  |  |  |
| - Buffer ATL concentrate |  |  |  |
| - Buffer AL concentrate |  |  |  |
| QIAgen Deparaffinisation Solution (Cat NO 19093) |  |  |  |
| *Other?* |  |  |  |

11. What proportion (%) of samples will have to repeat **stage 1** due to error? *Enter percentage* %

# Stage 2. Sample PREPARATION

12. Please indicate the **staff** responsible for the tasks detailed below. If this can vary depending on availability/ case complexity, please indicate the grade of staff most likely to be responsible. Please also indicate the time staff is actively involved:

| Staff: *Choose an item*  Grade: *Choose an item* | |
| --- | --- |
| Task | Staff time (min) |
| Identify the samples that require testing. Fill in the W307 Cobas Run sheet using lab number and patient's name. |  |
| Quantify the samples to be tested using the NanoDrop 2000. When possible dilute samples using DNA SD reagent into a new and appropriately labelled tube, with lab number and patient's surname. |  |

##### Equipment

13. Please provide details of the pieces of **equipment** used to complete the tasks in **stage 2**. Some of these items have already been entered for you, based on the Cobas 4800 SOP. If additional equipment is required, please add an additional row to the table and complete as appropriate:

| Type of equipment | Quantity required | Equipment cost (new) (£) | VAT included? | Annual maintenance cost (£)* | Predicted lifetime (years) |
| --- | --- | --- | --- | --- | --- |
| NanoDrop 2000 |  |  | Yes No |  |  |
| *Other?* |  |  | Yes No |  |  |

** If applicable*

14. Are these pieces of equipment used entirely for real-time PCR?

Yes (Go to **Q17**)

No (Go to **Q15**)

15. If **No**, which pieces of equipment are used elsewhere in the laboratory? List pieces of equipment

16. What proportion (%) of their time or capacity is for real-time PCR? Enter percentage %

17. Are there any pieces of equipment that are used in **stage 2** which require samples to be batched together, rather than being processed one sample at a time?

Yes (Go to **Q18**)

No (Go to **Q20**)

18. If **Yes**, which pieces of equipment require samples to be batched together? List pieces of equipment

19. What would the standard batch size be? Enter batch size

##### Consumables

20. Please list the **consumables** used to complete the tasks in **stage 2**. If needed, add additional rows to the table and complete as appropriate:

| Type of consumable | Overall cost (e.g. £10 for a box of consumables X) | Number of units (e.g. 100 in one box) | Number of units used per sample (e.g. 2) |
| --- | --- | --- | --- |
| DNA SD reagent |  |  |  |
| Labelled tubes |  |  |  |
| *Other?* |  |  |  |

21. What proportion (%) of samples will have to repeat **stage 2** due to error? *Enter percentage* %

# Stage 3. REAGENT preparation

22. Please indicate the **staff** responsible for the tasks detailed below. If this can vary depending on availability/ case complexity, please indicate the grade of staff most likely to be responsible. Please also indicate the time staff is actively involved:

| Staff: *Choose an item*  Grade: *Choose an item* | |
| --- | --- |
| Task | Staff time (min) |
| Calculate the volume of reagents needed for the number of samples, including controls, to be tested. |  |
| Prepare and label three eppendorfs for all three the reaction mixes and add the calculated volumes of MMX and MGAC. Vortex all tubes to assure adequate mixing. |  |

##### Equipment

23. Please provide details of the pieces of **equipment** used to complete the tasks in **stage 3**. Some of these items have already been entered for you, based on the Cobas 4800 SOP. If additional equipment is required, please add an additional row to the table and complete as appropriate:

| Type of equipment | Quantity required | Equipment cost (new) (£) | VAT included? | Annual maintenance cost (£)* | Predicted lifetime (years) |
| --- | --- | --- | --- | --- | --- |
| Vortexer |  |  | Yes No |  |  |
| *Other?* |  |  | Yes No |  |  |

** If applicable*

24. Are these pieces of equipment used entirely for real-time PCR?

Yes (Go to **Q17**)

No (Go to **Q15**)

25. If **No**, which pieces of equipment are used elsewhere in the laboratory? List pieces of equipment

26. What proportion (%) of their time or capacity is for real-time PCR? Enter percentage %

27. Are there any pieces of equipment that are used in **stage 3** which require samples to be batched together, rather than being processed one sample at a time?

Yes (Go to **Q28**)

No (Go to **Q30**)

28. If **Yes**, which pieces of equipment require samples to be batched together? List pieces of equipment

29. What would the standard batch size be? Enter batch size

##### Consumables

30. Please list the **consumables** used to complete the tasks in **stage 3**. Some of these consumables have already been entered for you, based on the Cobas 4800 SOP. If additional consumables are required, please add an additional row to the table and complete as appropriate:

| Type of consumable | Overall cost (e.g. £10 for a box of consumables X) | Number of units (e.g. 100 in one box) | Number of units used per sample (e.g. 2) |
| --- | --- | --- | --- |
| COBAS 4800 Mutation Kit Reagents. | *Please specify when costs differ between the EGFR, BRAF and KRAS Mutation Kits* |  |  |
| - MMX-1 |  |  |  |
| - MMX-2 |  |  |  |
| - MMX-3 |  |  |  |
| - MGAC |  |  |  |
| Eppendorfs |  |  |  |
| *Other?* |  |  |  |

31. What proportion (%) of samples will have to repeat **stage 3** due to error? *Enter percentage* %

# Stage 4. RUN PREPARATION

32. Please indicate the **staff** responsible for the tasks detailed below. If this can vary depending on availability/ case complexity, please indicate the grade of staff most likely to be responsible. Please also indicate the time staff is actively involved:

| Staff: *Choose an item*  Grade: *Choose an item* | |
| --- | --- |
| Task | Staff time (min) |
| Add reaction mix to each reaction well of the microwell plate (AD-plate) that is needed for the run. Add Mutant Control to wells and mix well. Add Negative Control (Standard Diluent) to wells and mix well. Add DNA specimen to wells and mix well. Repeat this procedure until all DNA samples are loaded into the microwell plate |  |
| Cover the microwell plate with sealing film. Use the sealing film applicator to seal the film firmly to the microwell plate. Centrifuge briefly to ensure all liquid is collected at the bottom of each well before starting PCR. |  |

##### Equipment

33. Please provide details of the pieces of **equipment** used to complete the tasks in **stage 4**. Some of these items have already been entered for you, based on the Cobas 4800 SOP. If additional equipment is required, please add an additional row to the table and complete as appropriate:

| Type of equipment | Quantity required | Equipment cost (new) (£) | VAT included? | Annual maintenance cost (£)* | Predicted lifetime (years) |
| --- | --- | --- | --- | --- | --- |
| Centrifuge |  |  | Yes No |  |  |
| Pipette |  |  | Yes No |  |  |
| *Other?* |  |  | Yes No |  |  |

** If applicable*

34. Are these pieces of equipment used entirely for real-time PCR?

Yes (Go to **Q27**)

No (Go to **Q25**)

35. If **No**, which pieces of equipment are used elsewhere in the laboratory? List pieces of equipment

36. What proportion (%) of their time or capacity is for real-time PCR? Enter percentage %

37. Are there any pieces of equipment that are used in **stage 4** which require samples to be batched together, rather than being processed one sample at a time?

Yes (Go to **Q38**)

No (Go to **Q40**)

38. If **Yes**, which pieces of equipment require samples to be batched together? List pieces of equipment

39. What would the standard batch size be? Enter batch size

##### Consumables

40. Please list the **consumables** used to complete the tasks in **stage 4**. Some of these consumables have already been entered for you, based on the Cobas 4800 SOP. If additional consumables are required, please add an additional row to the table and complete as appropriate:

| Type of consumable | Overall cost (e.g. £10 for a box of consumables X) | Number of units (e.g. 100 in one box) | Number of units used per sample (e.g. 2) |
| --- | --- | --- | --- |
| Cobas 4800 EGFR Mutation kit | *Please specify when costs differ between the EGFR, BRAF and KRAS Mutation Kits* |  |  |
| - Reaction mix |  |  |  |
| - Mutant Control |  |  |  |
| - Negative Control (Standard Diluent) |  |  |  |
| Cobas microwell plate |  |  |  |
| - Sealing film |  |  |  |
| - Sealing film applicator |  |  |  |
| *Other?* |  |  |  |

41. What proportion (%) of samples will have to repeat **stage 4** due to error? *Enter percentage* %

# Stage 5. COBAS 4800 - Sequencing

42. Please indicate the **staff** responsible for the tasks detailed below. If this can vary depending on availability/ case complexity, please indicate the grade of staff most likely to be responsible. Please also indicate the time staff is actively involved:

| Staff: *Choose an item*  Grade: *Choose an item* | |
| --- | --- |
| Task | Staff time (min) |
| Switch on the computer and the instrument. Open the Cobas software. Enter your login and password and log on. Set up the microwell plate. |  |
| Scan the microwell plate barcode. Enter the barcode of the DNA sample preparation kit. Scan the barcode of the EGFR Mutation Test kit. Type in the number of specimens. Enter the sample ID barcodes. |  |
| Load the microwell plate in the Cobas analyser. Start the run. When completed unload and discard the microwell plate. |  |

##### Equipment

43. Please provide details of the pieces of **equipment** used to complete the tasks in **stage 5**. Some of these items have already been entered for you, based on the Cobas 4800 SOP. If additional equipment is required, please add an additional row to the table and complete as appropriate:

| Type of equipment | Quantity required | Equipment cost (new) (£) | VAT included? | Annual maintenance cost (£)* | Predicted lifetime (years) |
| --- | --- | --- | --- | --- | --- |
| Barcode scanner |  |  | Yes No |  |  |
| Cobas 4800 analyser |  |  | Yes No |  |  |
| Computer |  |  | Yes No |  |  |
| *Other?* |  |  | Yes No |  |  |

** If applicable*

44. Are these pieces of equipment used entirely for real-time PCR?

Yes (Go to **Q37**)

No (Go to **Q35**)

45. If **No**, which pieces of equipment are used elsewhere in the laboratory? List pieces of equipment

46. What proportion (%) of their time or capacity is for real-time PCR? Enter percentage %

47. Are there any pieces of equipment that are used in **stage 5** which require samples to be batched together, rather than being processed one sample at a time?

Yes (Go to **Q48**)

No (Go to **Q50**)

48. If **Yes**, which pieces of equipment require samples to be batched together? List pieces of equipment

49. What would the standard batch size be? Enter batch size

##### Consumables

50. Please list the **consumables** used to complete the tasks in **stage 5**. Some of these consumables have already been entered for you, based on the Cobas 4800 SOP. If additional consumables are required, please add an additional row to the table and complete as appropriate:

| Type of consumable | Overall cost (e.g. £10 for a box of consumables X) | Number of units (e.g. 100 in one box) | Number of units used per sample (e.g. 2) |
| --- | --- | --- | --- |
|  |  |  |  |
|  |  |  |  |

51. What proportion (%) of samples will have to repeat **stage 5** due to error? *Enter percentage* %

# STAGE 6. Interpretation and reporting

52. Please indicate the **staff** responsible for the tasks detailed below. If this can vary depending on availability/ case complexity, please indicate the grade of staff most likely to be responsible. Please also indicate the time staff is actively involved:

| Task | Staff type | Staff grade | Staff time (min) |
| --- | --- | --- | --- |
| Review results and ensure that the run is valid. If the run is invalid, report to a senior member of staff. | *Choose an item* | *Choose an item* |  |
| Accept results and export a complete run. Print the report and store with the W307 run sheet. | *Choose an item* | *Choose an item* |  |
| Interpret the results for each sample and re-transcribe the results onto the record sheet for the corresponding patient. | *Choose an item* | *Choose an item* |  |
| The report is reviewed and counter-signed by senior clinical scientist before being sent away. | *Choose an item* | *Choose an item* |  |

##### Equipment

53. Please provide details of the pieces of **equipment** used to complete the tasks in **stage 6**. If additional equipment is required, please add an additional row to the table and complete as appropriate:

| Type of equipment | Quantity required | Equipment cost (new) (£) | VAT included? | Annual maintenance cost (£)* | Predicted lifetime (years) |
| --- | --- | --- | --- | --- | --- |
|  |  |  | Yes No |  |  |
|  |  |  | Yes No |  |  |

** If applicable*

54. Are these pieces of equipment used entirely for real-time PCR?

Yes (Go to **Q57**)

No (Go to **Q55**)

55. If **No**, which pieces of equipment are used elsewhere in the laboratory? List pieces of equipment

56. What proportion (%) of their time or capacity is for real-time PCR? Enter percentage %

57. Are there any pieces of equipment that are used in **stage 6** which require samples to be batched together, rather than being processed one sample at a time?

Yes (Go to **Q58**)

No (Go to **Q60**)

58. If **Yes**, which pieces of equipment require samples to be batched together? List pieces of equipment

59. What would the standard batch size be? Enter batch size

##### Consumables

60. Please list the **consumables** used to complete the tasks in **stage 6**. If additional consumables are required, please add an additional row to the table and complete as appropriate:

| Type of consumable | Overall cost (e.g. £10 for a box of consumables X) | Number of units (e.g. 100 in one box) | Number of units used per sample (e.g. 2) |
| --- | --- | --- | --- |
|  |  |  |  |
|  |  |  |  |

61. What proportion (%) of samples will have to repeat **stage 6** due to error? *Enter percentage* %

# ADDITIONAL STAGE (IF REQUIRED)

62. Please indicate the **staff** responsible for the tasks detailed below. If this can vary depending on availability/ case complexity, please indicate the grade of staff most likely to be responsible. Please also indicate the time staff is actively involved:

| Task | Staff type | Staff grade | Staff time (min) |
| --- | --- | --- | --- |
|  |  |  |  |
|  |  |  |  |

##### Equipment

63. Please provide details of the pieces of **equipment** used to complete the tasks in **this additional stage**. If additional equipment is required, please add an additional row to the table and complete as appropriate:

| Type of equipment | Quantity required | Equipment cost (new) (£) | VAT included? | Annual maintenance cost (£)* | Predicted lifetime (years) |
| --- | --- | --- | --- | --- | --- |
|  |  |  | Yes No |  |  |
|  |  |  | Yes No |  |  |

** If applicable*

64. Are these pieces of equipment used entirely for real-time PCR?

Yes (Go to **Q67**)

No (Go to **Q65**)

65. If **No**, which pieces of equipment are used elsewhere in the laboratory? List pieces of equipment

66. What proportion (%) of their time or capacity is for real-time PCR? Enter percentage %

67. Are there any pieces of equipment that are used in **this additional stage** which require samples to be batched together, rather than being processed one sample at a time?

Yes (Go to **Q68**)

No (Go to **Q70**)

68. If **Yes**, which pieces of equipment require samples to be batched together? List pieces of equipment

69. What would the standard batch size be? Enter batch size

##### Consumables

70. Please list the **consumables** used to complete the tasks in **this additional stage**. If additional consumables are required, please add an additional row to the table and complete as appropriate:

| Type of consumable | Overall cost (e.g. £10 for a box of consumables X) | Number of units (e.g. 100 in one box) | Number of units used per sample (e.g. 2) |
| --- | --- | --- | --- |
|  |  |  |  |
|  |  |  |  |

71. What proportion (%) of samples will have to repeat **this additional stage** due to error? *Enter percentage* %

# MISCELLANEOUS

72. Are there any other costs that are incurred in undertaking real-time PCR which are not specific to any of the above stages e.g. IT equipment and stationery? If needed, add additional rows to the table and complete as appropriate:

| Type of equipment | Quantity required | Equipment cost (new) (£) | VAT included? | Annual maintenance cost (£)* | Predicted lifetime (years) |
| --- | --- | --- | --- | --- | --- |
| Cobas software |  |  | Yes No |  |  |
| *Other?* |  |  | Yes No |  |  |

** If applicable*
